# *De-novo* assembly and characterization of the transcriptome of *Metschnikowia fructicola* reveals differences in gene expression following interaction with *Penicillium digitatum* and grapefruit peel

**DOI:** 10.1186/1471-2164-14-168

**Published:** 2013-03-12

**Authors:** Vera Hershkovitz, Noa Sela, Leena Taha-Salaime, Jia Liu, Ginat Rafael, Clarita Kessler, Radi Aly, Maggie Levy, Michael Wisniewski, Samir Droby

**Affiliations:** 1 Department of Postharvest and Food Sciences, ARO, the Volcani Center, Bet Dagan, 50250, Israel; 2 Department of Plant Pathology and Weed Research, ARO, the Volcani Center, Bet Dagan, 50250, Israel; 3 Department of Plant Pathology and Weed Research, the Volcani Center, Newe-Yaar Research Center, Ramat Yeshai, 30095, Israel; 4 Department of Plant Pathology and Microbiology, the Robert H. Smith Faculty of Agriculture, Food and Environment, the Hebrew University of Jerusalem, Revovot, 76100, Israel; 5 U.S. Department of Agriculture-Agricultural Research Service (USDA-ARS), Appalachian Fruit Research Station, Kearneysville, WV, 25430, USA

**Keywords:** Antagonist-fruit interaction, Antagonist-pathogen interaction, Biological agent, RNA-seq

## Abstract

**Background:**

The yeast *Metschnikowia fructicola* is an antagonist with biological control activity against postharvest diseases of several fruits. We performed a transcriptome analysis, using RNA-Seq technology, to examine the response of *M. fructicola* with citrus fruit and with the postharvest pathogen, *Penicillium digitatum*.

**Results:**

More than 26 million sequencing reads were assembled into 9,674 unigenes. Approximately 50% of the unigenes could be annotated based on homology matches in the NCBI database. Based on homology, sequences were annotated with a gene description, gene ontology (GO term), and clustered into functional groups. An analysis of differential expression when the yeast was interacting with the fruit vs. the pathogen revealed more than 250 genes with specific expression responses. In the antagonist-pathogen interaction, genes related to transmembrane, multidrug transport and to amino acid metabolism were induced. In the antagonist-fruit interaction, expression of genes involved in oxidative stress, iron homeostasis, zinc homeostasis, and lipid metabolism were induced. Patterns of gene expression in the two interactions were examined at the individual transcript level by quantitative real-time PCR analysis (RT-qPCR).

**Conclusion:**

This study provides new insight into the biology of the tritrophic interactions that occur in a biocontrol system such as the use of the yeast, *M. fructicola* for the control of green mold on citrus caused by *P. digitatum*.

## Background

Considerable research has been devoted in the past two decades to investigate the use of naturally occurring yeasts for managing postharvest diseases on a variety of fruits and vegetables [1,2]. At present, only two commercial products are available for postharvest use, each having a very small market share of the collective technologies used to manage postharvest diseases. Biosave (*Pseudomonas syringae* Van Hall) used for the control of pome fruit, sweet potato, and potato diseases [3], and “Shemer” (*Metschnikowia fructicola)*[4] used commercially for the control of sweet potato and carrot storage diseases [4], strawberry [5], apple [6] and citrus fruit [7].

Knowledge about mechanisms of action of yeast biocontrol agents used to manage postharvest diseases is relatively superficial because appropriate methods to study microbial interactions in wounds of fruit has been lacking. Various mechanisms, however, have been described, including antibiosis, production of lytic enzymes, parasitism, induction of host resistance, and competition for limiting nutrients and space [2,8-10]. Results described in these studies are mostly correlative and provide indirect evidence for possible involvement of one or more of these components in the mechanism of action. This may be due to the lack of an understanding of the key biochemical and molecular processes occurring within tritrophic interactions (host-pathogen-biocontrol agent) that define the effectiveness of a biocontrol system.

A characteristic response of fruit to pathogen attack or application of a biocontrol agent is the activation of defence responses, including production of inhibitors of cell wall-degrading enzymes of the pathogen, activity of antifungal compounds (such as phenolic compounds and phytoalexins), active oxygen species, and reinforcement of the cell wall of the host [9]. In relation to yeast biocontrol agents, induction of resistance in fruit tissue has been reported to involve, to a greater or lesser extent, the active elicitation of all of the above responses. Hershkovitz et al. [11] recently reported the first molecular study of the response of citrus fruit to the application of the yeast biocontrol agent, *M. fructicola*. Results indicated that significant changes in gene expression occur in grapefruit wounded tissue in response to the application of *M. fructicola* including changes in a variety of genes involved in response to biotic and abiotic stresses, signaling, defence and secondary metabolism. These findings imply that complex biochemical and molecular processes are involved in the reaction of fruit host tissue to the introduction of yeast cells, which may have the potential to influence the efficacy of the biocontrol agent.

Castoria et al. [12] indicated that the ability to tolerate high levels of reactive oxygen species (ROS) produced in fruit tissue in response to wounding is an essential characteristic of effective yeast antagonists. Macarisin et al. [7] demonstrated that the antagonistic yeasts, *Candida oleophila* and *M. fructicola*, actually induce defence-related oxidative responses in intact fruit exocarp tissues and within wounds of apple and citrus, generating greater levels of super oxide anion (O_2_−) and hydrogen peroxide (H_2_O_2_). Torres et al. [13] reported that, in addition to nutrient competition, the biocontrol activity of *Pantoea agglomerans* could be attributed to the ability of the antagonist to induce an oxidative response in orange fruit peel tissue by triggering H_2_O_2_ production, superoxide dismutase, and catalase activities. In another study, transient exposure of the yeast *M. fructicola* to a sublethal heat or oxidative stress increased overall abiotic stress tolerance, providing cross-protection to a range of other more severe abiotic stresses [6]. The study showed that higher levels of trehalose, induced by exposure to a sub-lethal abiotic stress, was associated with an increase in ROS scavenging, general stress tolerance, and more rapid growth in apple wounds, collectively resulting in improved biocontrol activity.

Larralde-Corona et al. [14] investigated the effect of the exposure of the yeast *Pichia guilliermondii* to *Penicillium digitatum* on yeast gene expression by analyzing expressed sequencing tags (ESTs) obtained by subtractive suppressive hybridization (SSH). The yeast was exposed to starvation by carbon source, actively growing mycelium of *P. digitatum* separated from yeast cells with membrane and induction by fungal cell walls. Only one EST, associated with energy metabolism, was obtained in response to the limited carbon source, seven ESTs in the separated membrane system, all relating to metabolic networks such as: energy, nitrogen, cell cycle, ABC transporters, response to stress and one unknown sequence. The exposure of the yeast cells to fungal cell walls produced the highest number of ESTs, a total of 22, including all the metabolic networks mentioned above plus ESTs associated with signal transduction.

Compared to the information available about the response of host tissue to the introduction of yeast biocontrol agents, little is known about global molecular changes taking place in yeast cells in response to an interaction with either fruit tissue or a pathogen's mycelia. In the current study, we characterized transcriptional changes in *M. fructicola* cells following their interaction with grapefruit peel tissue and mycelium of *P. digitatum* using next generation sequencing technology for RNA (RNA-Seq) on the Illumina platform. Along with *de-novo* assembly and characterization of the transcriptome, analysis of global patterns of gene expression and functional categorization were performed.

## Results

### Illumina sequencing, assembly, and annotation

**Figure 1:**
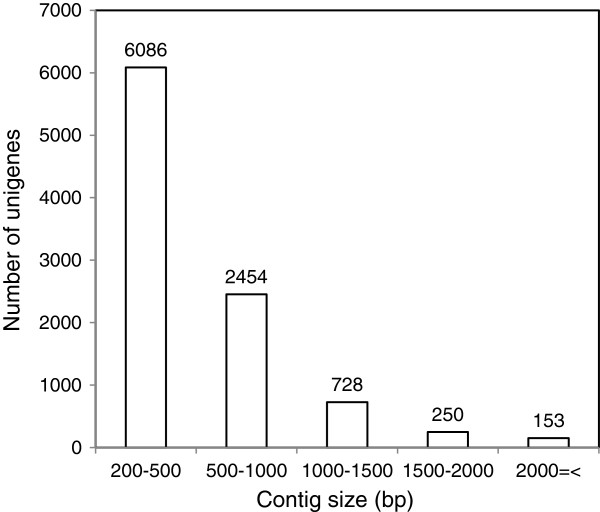
**Size distribution of the *Metschnikowia fructicola *cells unigenes.**

Transcriptome analysis of *M. fructicola* utilizing Illumina sequencing generated a total of 26,829,178 reads. The raw reads were assembled into 15,803 contigs by Trinity software with an average size of 490.2 bp and median of 349 bp. The sequences were determined to represent 9684 unigenes with an average length of 546.2 bp and median of 385 bp. The size distribution is shown in Figure 1. To annotate these genes, reference sequences were searched using BLASTX within the non-redundant (nr) NCBI nucleotide database using a cut-off E-value of 10^-3^ (the sequences of the unigenes are found in Additional file 1 in fasta format). A total of 2452 genes (25.3% of all gene sequences) could not be annotated based on matches in the NCBI database (the full annotations of *M. fructicola* unigenes is found in Additional file 2). A high level (75%) of transcripts from the individual libraries could be mapped to the assembled transcriptome using bowtie software. These transcripts were blasted against the *M. fructicola* draft genome (available under genbank accession number ANFW01000000) with cutoff e-value of 1e-50 and identity >85%. According to this analysis 9631 unigenes (99.4%) could be aligned to the genome, indicating high accuracy of our assembly (mapping information is found in Additional file 3). The remaining 53 unigenes could be a result of erroneous assembly.

**Figure 2:**
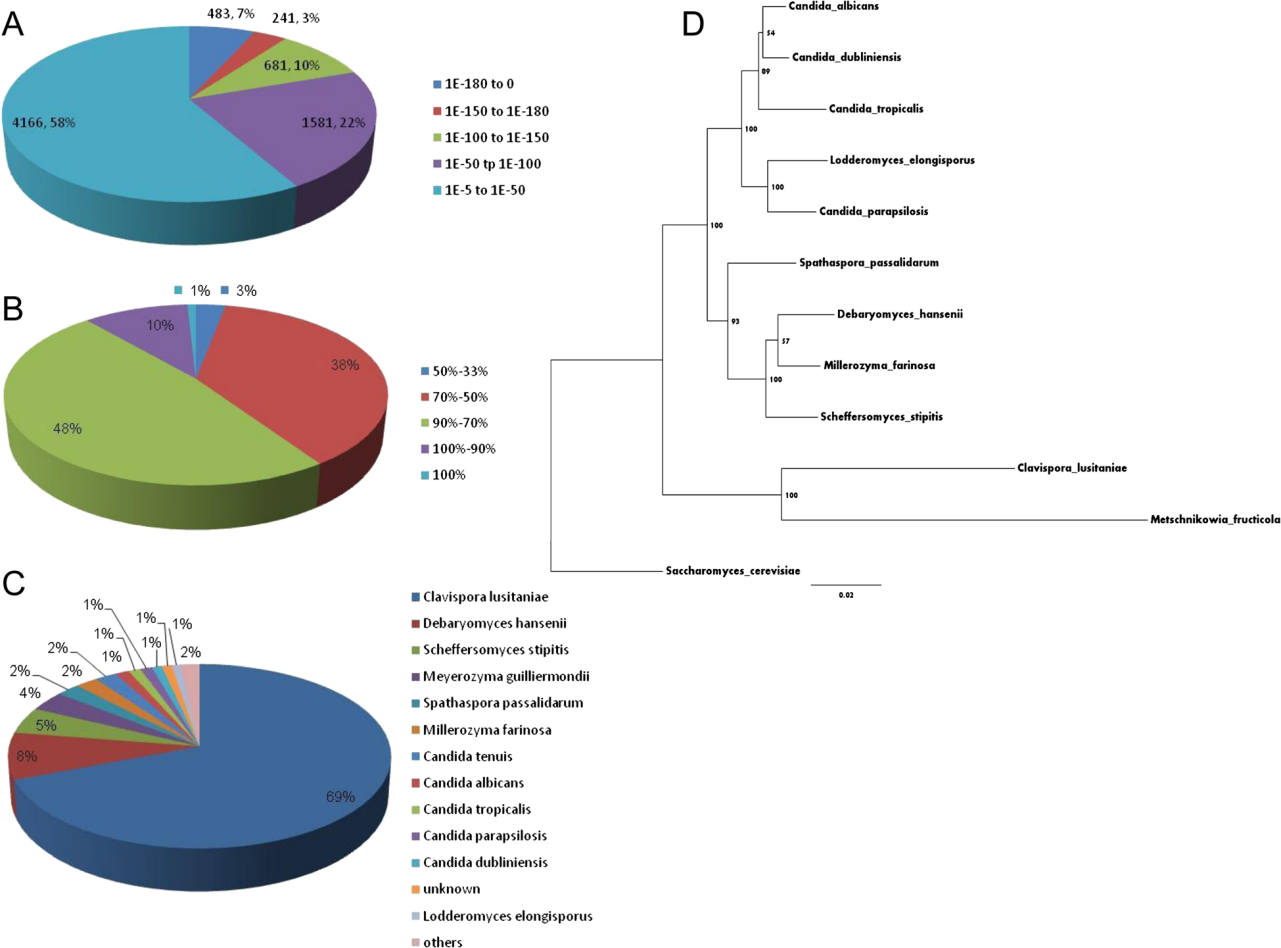
**Homology analysis of the *Metschnikowia fructicola *trasncriptome.** All distinct gene sequences that had BLAST annotations within the nr database with a cut-off E-value < =10-5 were analyzed for **A**, E-value distribution **B**, similarity distribution **C**, species distribution and **D**, phylogenetic tree based on ribosomal RNA large subunit sequences.

As shown in Figure 2, 42% of sequences had significant homology matches (< 1.0E10^− 50^) in the NCBI database, while 58% of the sequences had matches ranging between 1.0E10^-3^ and 1.0E10^-50^. The similarity distribution showed that 11% of the sequences had > 90% homology, followed by 86% of the sequences ranging between 50 to 90% homology. Only 3% of the sequences had homology lower than 50% (Figure 2B). Species specific distribution indicated that 69% of the sequences matched gene sequences of the yeast *Clavispora lustiniae*, followed by other yeasts *Debar-yomyces hansenii* (8%), *Scheffersomyces stiptis* (5%), *Meyerozyma guilliermondii* (4%), *Spathaspora passa-lidarum*, *Millerozyma farinose* and *Candida tenuis* (2% each) (Figure 2C). The phylogenetic analyses presented in Figure 2D shown close sequence similarity between *M. fructicola* and *C. lustiniae* (Figure 2D).

### Classification of gene ontology (GO)

**Figure 3:**
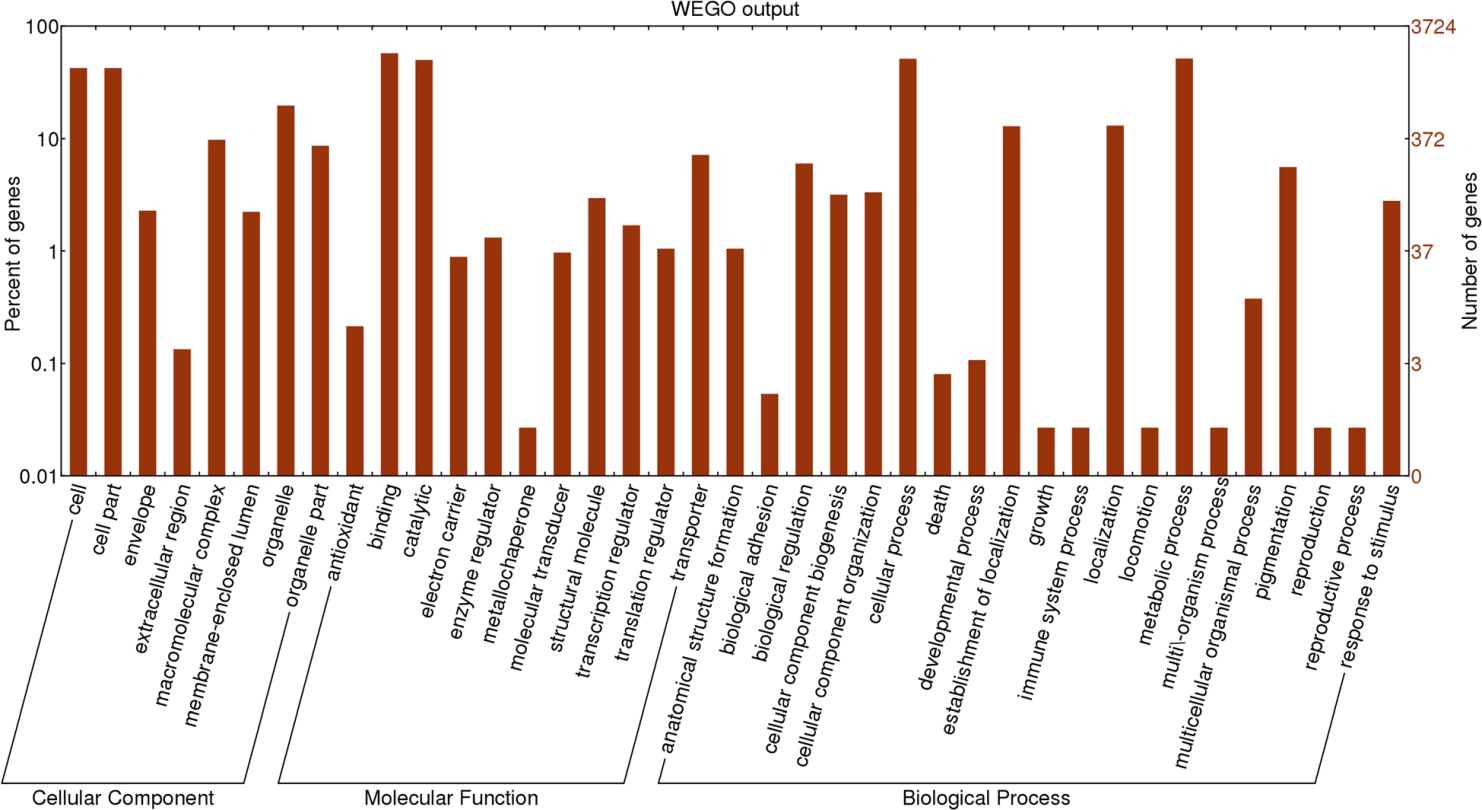
**Gene ontology (GO) classification of the *Metschnikowia fructicola *unigenes.** Out of 9674 unigenes, 4126 sequences were annotated within GO database into three main categories: cellular component, biological process, molecular function and. Y-axis indicate the number of unigenes while the x-axis indicate the GO category.

To classify the functions of *M. fructicola* unigenes, we searched the Gene Ontology (GO) database and used the WEGO tool (web gene ontology annotation Plotting) for plotting GO annotation results (http://wego.genomics.org.cn). A total of 4126 sequences out of 9674 (57.6% of annotated sequences) were categorized into 43 functional groups (Figure 3). In each of the three main categories (biological process, cellular component and molecular function) of the GO classification the 'Cell and Cell part', 'Antioxidant and Binding' and 'Metabolic and cellular processes' terms were the most dominant. In contrast, 'metallochapreon', and 'rhythmic process' were least abundant.

### Transcription profiles

**Figure 4:**
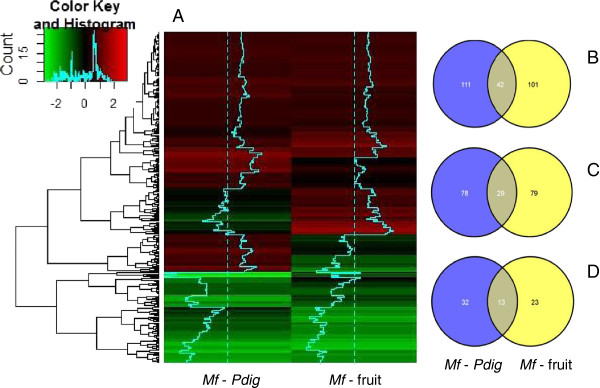
**Expression profiles of *Metschnikowia fructicola. *** Two-dimensional hierarchical cluster heat map of differential expression profiles for 254 genes from *Mf-Pdig* and *Mf*-fruit interactions (**A**). Genes with increased expression are shown in red, genes that were downregulated are colored green, and those showing no change relative to expression in are in brown. Color intensity is proportional to differential expression (log2 Fold Change). Venn diagram showing all differential expressed (**B**), up regulated (**C**) and down regulated (**D**) genes in *Mf-Pdig* and *Mf*-fruit interactions. The number of genes commonly expressed in *Mf-Pdig* and *Mf*-fruit interactions is provided in the overlapping portions of the circles.

RNA-seq technology is a sensitive and reliable tool to study transcriptional changes. Using this technique we identified 153 genes differentially expressed (DE) with at least a 1.8-fold change in yeast cells in response to yeast 24 h after interaction with *P. digitatum* (antagonist-pathogen: *Mf-Pdig*). Among these, 105 were upregulated and 45 were downregulated. In the *Mf*-fruit interaction, 143 genes had differential expression where 108 were upregulated and 36 were downregulated, while 42 genes were regulated in both interactions. DE genes in either *Mf-Pdig* and *Mf*-fruit interactions or specific to one of these interactions are shown in a two-dimensional hierarchical cluster heat map and Venn diagram (Figure 4) Additional file 4.

### Functional analysis of differentially expressed genes

**Figure 5:**
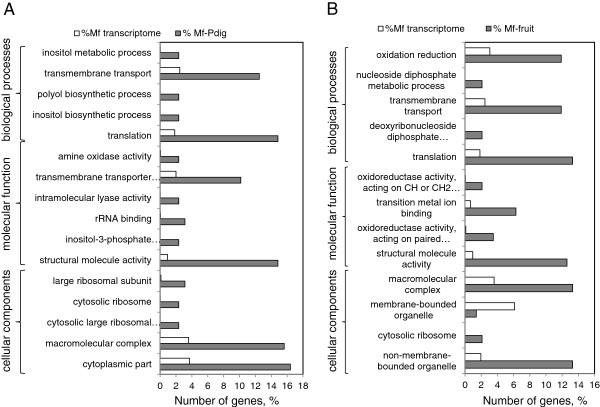
**Gene ontology (GO) classification of the *Metschnikowia fructicola *differentially expressed genes.** Summary of GO categories for the all transcribed genes in *M. fructicola* cells (*Mf* transcriptome) and differentially expressed genes in *M. fructicola* cells during interaction with *P.digitatum* (*Mf-Pdig*) (**A**) and during interaction with fruit (*Mf*-fruit) (**B**).

**Figure 6:**
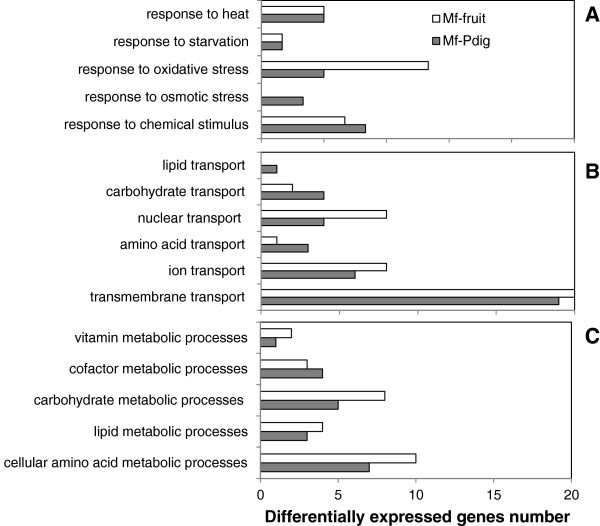
**The differentially expressed genes in *Metschnikowia fructicola *cells following interaction with *Penicillium digitatum *(*Mf-Pdig*) or with grapefruit (*Mf*-fruit) related to response to stress and to stimulus (A), transport functions (B) and metabolic processes (C).**

All differentially regulated genes were organized in functional categories based on their gene ontology (GO) terms (http://www.blast2go.com/b2glaunch) and were members of at least one of the three principal GO branches: biological process, molecular function, and cellular component (Figure 5). All 16 functional groups were found to be over-expressed in *Mf-Pdig* interaction compared to all transcribed genes of *M. fructicola* controls (Figure 5A). We focused our analyses on “biological process” categories. Those categories were organized in three groups: response to stress and stimulus transport (Figure 6A), transport (Figure 6B) and metabolic processes (Figure 6C).

The induced genes in the category of response to stress in *Mf-Pdig* interaction are mainly involved in multidrug resistance and resistance to singlet oxygen species (*YHK, YOR1, SNQ2* and *STB5*) and in oxidation-reduction activity in the *Mf*-fruit interaction (*MET10, MET5, RNR2*, *HEM13, JLP1, FET3, AFG1* and *PRX1*) (Figure 6A; Additional file 5). The genes encoding enzymes involved in response to heat stress were, such as heat shock proteins HSP78, HSP40, HSP104 and SGT2, were repressed, in both of interactions.

Transport genes involved in iron transport and uptake (*FTR1, FET3* and *SIT2*), zinc transport (*ZTR2* and *ZTR3*) and SulP anion (*SUL1*) were upregulated in the *Mf*-fruit interaction while in the *Mf-Pdig* interaction, ammonium permease (*MEP2*), inorganic phosphate (Pi) transporter and low-affinity manganese transporter (*PHO84*) were induced (Figure 6B; Additional file 6).

A glucose sensor (*SNF3*) and two glucose transporter (*HXT* 6 and 4) genes were induced in the *Mf*-*Pdig* interaction. A group of genes associated with transmembrane transport, such as general amino acid permease (*GAP1*, myo-inositol transporter (*ITP2*), oligopeptide transporter (*OPT2*), a permease (*GIT1*), which mediates uptake of glycerophosphoinositol and glycerophosphocholine as sources for inositol and phosphate, an a sugar transporter (*HXT3*), were induced in both interactions.

Functional analysis of the metabolic processes category included lipid, cofactor and cellular amino acid metabolic process annotations. Many genes involved in the ergosterol-biosynthesis pathway (*ERG1, ERG 11, ERG5, PSD* and *DAP1*) were up-regulated in the *Mf-*fruit interaction (Additional file 7).

Many genes involved in amino acid metabolism (*AGP3, GAP1, UGA1, CPA2, MAE1, CAR2 and FMS1*) were up-regulated in the *Mf-Pdig* interaction. In the *Mf*-fruit interaction, two groups of genes involved in cellular amino acid metabolism were found to be enriched: one up-regulated (*CAP1, CHI, LAP3, ARO1, LIA1* and *PDC1*) and the second, down-regulated (*MIS1, OXP1, ALD3, CAR1* and *GCV1*) (Additional file 7).

### Real time PCR (qRT-PCR) analysis

**Figure 7:**
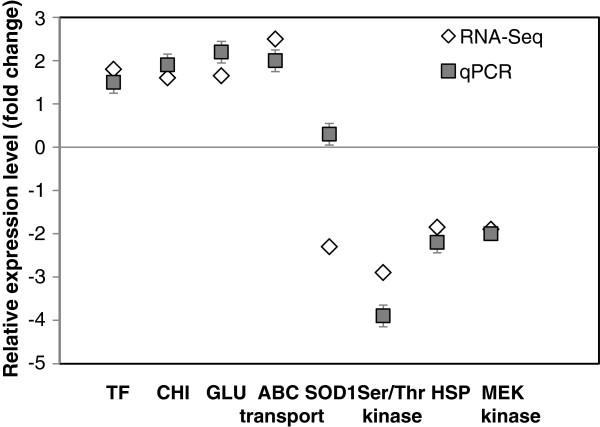
**Comparison results of RNA-seq (◊) and qRT-PCR (■ ).** Expression profiling of TF, CHI, GLU, ABC transporter, SOD1, Ser/Thr kinase, HSP, MEK kinase genes were examined by RNA-Seq and qPCR in genes in *M. fructicola* cells during interaction with *P.digitatum* (*Mf-Pdig*). Vertical lines represent standard error for an average of three biological replicates.

**Figure 8:**
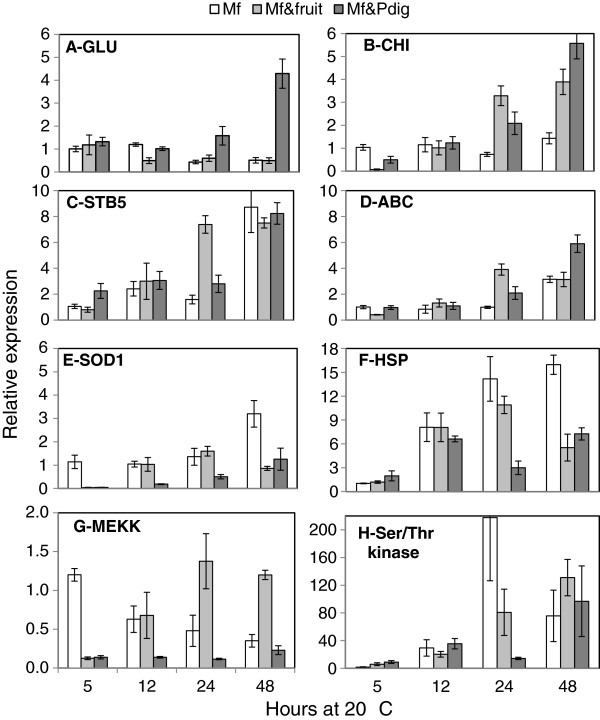
**The differentially expressed genes profile detected by qRT-PCR.** qRT-PCR was performed for of *GLU* (**A**), *CH*I (**B**), *STB5* transcription factor (**C**), *ABC* transporter (**D**), *SOD* (**E**), *HSP* (**F**), *MEKK* (**G**) and *Ser/Thr kinase* (**H**) in *M. fructicola* cells (*Mf*), *M. fructicola* cells following interaction with *P. digitatum, (Mf-Pdig) and M. fructicola* cells following interaction with grapefruit (*Mf*-fruit) at 5, 12, 24 and 48 h after interaction. Values have been normalized to *Mf* at 5 h, arbitrarily set to 1. Vertical lines represent standard error for an average of three biological replicates.

To confirm the expression profiles obtained from the RNA-seq expression data, RT-qPCR analysis was carried out for 8 genes selected on the basis of their biological significance: genes involved in the degradation of fungal cell wall, such as glucanse (*GLU*) and chitinase (*CHI*), were selected as were genes related to response to stresses, such as an *ABC* transporter, a transcription factor (*STB5*), a heat shock protein (*HSP-40*), superoxide dismutase (*SOD1*), a serine threonine (*Ser-Thr*) kinase, and a signal transducing *MEK* kinase. Although minor variation of transcription levels were obtained, 7 of the tested 8 genes correlated well with the fold-change, either up or down- obtained in the RNA-Seq analysis (analyzed the 24 h time point) (Figure 7). Time points included in the RT-qPCR analysis were 5, 12, 24 and 48 h after interaction of the yeast cells with either grapefruit peel or *P. digitatum* mycelia (Figure 7). Expression level of the *SOD1* gene in RT-qPCR was two-fold higher compare to the RNA-seq data set. Transcript levels of *GLU* were induced in *M. fructicola* cells after 24 and 48 h in the *Mf-Pdig* interaction (Figure 8A). *CHI* levels were up-regulated in both *Mf-Pdig* and *Mf*-fruit interactions after 24 h and 48 h (Figure 8B). *STB5* expression level displayed transient induction in both *Mf*-fruit and *Mf-Pdig* interactions at 24 h (Figure 8C), while the *Ser-Thr* kinase was transiently repressed in both *Mf*-fruit and *Mf-Pdig* interactions at 24 h (Figure 8H). The ABC transporter was induced in the *Mf-Pdig* interaction at 24 and 48 h, while in the *Mf*-fruit interaction it was induced only at 24 h (Figure 8D). Expression levels of *SOD1* and *MEKK* were repressed in the *Mf-Pdig* interaction at all-time points tested compared to control cells grown in NYDB media. In the Mf-fruit interaction these genes showed a transient induction at 12 and 24 h (Figure 8E, G). *HSP* transcript levels increased following contact with either the fruit peel or *P. digitatum* mycelia but were down-regulated in comparison to the control, *M. fructicola* cells grown in NYDB (Figure 8F).

## Discussion

Information about the interactions between yeast biocontrol agents, pathogens, and plant hosts (mainly harvested fruit) is needed to better understand biochemical and molecular processes involved in the biocontrol system [10,15]. In a previous study, we identified global changes in gene expression in grapefruit peel tissue following the application of the yeast *Metschnikowia fructicola*[11]. Marked changes in the profiles of genes expressed in fruit tissue were observed in response to application of yeast into wounded fruit tissue. In the present study, the main objective was to compare changes in gene expression in *M. fructicola* cells following the interaction of the yeast with either grapefruit peel tissue or *P. digitatum* mycelia.

Although the genome of *M. fructicola* has not been sequenced, de-novo assembly of the transcriptome of *M. fructicola* resulted in the identification of a total of 9674 unigenes, half of which could be annotated based on homology to genes in the NCBI database (Figures 1 and 2). As shown in Figure 2C, 69% of the unigene sequences identified in *M. fructicola* showed highest homology to *Clavispora lustiniae* genes*.* Thus the RNA-Seq based transcriptome analysis of *M. fructicola* generated a large number of newly identified yeast genes, providing a substantial contribution to the existing sequence database.

Using RNA-seq, we compared transcriptomes of *M. fructicola* after the yeast were exposed to either grapefruit peel tissue (*Mf-Pdig*) or after coming into direct contact with the fungal mycelia of *P. digitatum* (*Mf-Pdig*). Our analysis identified more than 250 genes that were differentially expressed and that may potentially affect the biocontrol activity of *M. fructicola* (Figure 4). The number of genes regulated in *Mf*-fruit interaction approximately same that regulated in *Mf-Pdig* and the overlap between the two interaction is only 16.5% of genes co-regulated.

### Stress related genes

Activation of stress-related genes in yeast biocontrol agents induced in response to the pathogen or plant host has been observed in several biocontrol systems [14,16,17]. In the current study, several genes involved in oxidation – reduction activity (GO: 0055114) and implicated in oxidative stress response, such as peroxidases and reductases, were up-regulated in the *Mf*-fruit interaction (Additional file 5). These results could be associated with the generation and detoxification of reactive oxygen species (ROS).

*M. fructicola* cells produce ROS as in response to contact with fruit tissue [7,11]. These studies indicate that ROS plays a major role in the initial response of yeast cells to host tissue and act as a signal, inducing an oxidative burst in the fruit tissue which subsequently leads to the induction of resistance mechanisms in the host tissue.

The time course study of superoxide dismutase (*SOD1*) gene expression showed a transient induction in *Mf*-fruit samples at 12 and 24 h (Figure 8E). These results provide further evidence that yeast cells are undergoing oxidative stress while in contact with fruit tissue and suggest the possibility of an adaptive response. In this regard, Liu et al. [6] demonstrated that a mild heat-shock pretreatment (30 min at 40°C) improved the tolerance of *M. fructicola* to subsequent high temperature (45°C, 20–30 min) and oxidative stress (0.4 mol L^-1^) and increased the biocontrol efficacy of the yeast. On the other hand, lower or unaltered expression levels of SOD (Figure 8E) and low levels of ROS in *Mf-Pdig* interaction (data not shown) indicate that in this type of interaction, yeast cells are not under oxidative stress and hence activation of protective systems against superoxide radicals is not necessary.

High levels of oxidative stress in microorganisms is accompanied by the induction of heat-shock proteins (HSP) in an attempt to ameliorate damage to proteins caused by ROS [18]. Induction of HSPs in response to biotic and abiotic stimuli is well documented where they assist in the folding and unfolding, and general stability, of various proteins. Production of HSPs in microorganisms is associated with various abiotic stresses, including oxidative stress [19]. In our present study, genes encoding HSP78 and HSP104 were down regulated in both types of interactions compared to the control (yeast grown in NYDB) (Additional file 5). However, the time course study of *HSP78* expression by RT-qPCR revealed significantly higher levels of transcript after 12 h, 24 and 48h in both interactions compared to its level at 5 h (Figure 8F). This result is surprising since it was anticipated that HSPs would be induced in yeast cells that are in contact with fruit peel tissue or with fungal mycelia considering the stressful environment it represents, such as desiccation due to the dryness of the fruit surface, ROS, and phytoalexins.

### Response to chemical stress

Genes belonged to plasma membrane ATP-binding cassette (ABC) transporters, such as *YHK8, YOR1* and *SNQ2,* were upregulated in the *MF-Pdig* interaction (Additional file 6). *YOR1* and *SNG2* genes were induced in *S. cerevisiae* following exposure to the monoterpene thymol [20]. The function of these transporters in *S. cerevisiae* and their possible role in yeasts tolerance to monoterpene have yet to be elucidated [21]. In *Pichia guilliermondii*, two ABC transporter related proteins involved in the secretion of toxic compounds were induced during its interaction with *P. digitatum*[14]. Many of the ABC transporters are reported to be tightly regulated by transcription factors [22]. In this regard, the current study showed that *STB5*, a transcription factor involved in regulating multidrug resistance and oxidative stress response, was transiently upregulated in the *Mf-Pdig* interaction at 24 h (Figure 8C). Induction of ABC transporters in *M. fructicola* cells may be needed for the yeast to tolerate exposure to fruit surface wounds or to mycelia where many potentially toxic substances (e.g. ROS, phytoalexines, toxins) may be present. This explanation may also be relevant to the ability of yeasts to tolerate high levels of ROS in apple fruit wounds which was suggested to be an essential characteristic of a successful biocontrol agent [12].

### Hydrolytic enzymes

Lytic enzymes, such as glucanases and chitinases, involved in degradation of fungal cell walls have been considered to play an important role in the biological control of postharvest pathogens [23-25]. Jijakli and Lepoivre [26] indicated that *P. guillermondii*, a yeast biocontrol agent, exhibits a high level of β-1,3-glucanase activity that could function to degrade the cell walls of postharvest pathogens. *P. membranefaciens* had higher β-1,3-glucanase and exochitinase activity but less endo-chitinase activity than *C. albidus* in the presence of fungal cell walls [27].

Several studies have demonstrated the ability of yeasts to produce and secrete glucanases and chitinases in culture medium amended with fungal cells walls [16,25,28]. The ability of yeasts to produce exo-ß-1,3-glucanase and chitinase has been suggested to play a role in the firm attachment of yeast cells to fungal hyphae and the partial degradation of fungal mycelia [1]. The current study clearly indicated that glucanase (*GLU*) and chitinase (*CHI*) genes are up regulated when yeast cells were in contact with *P. digitatum* mycelia (*Mf-Pdig*). When yeast cells were in contact with grapefruit peel tissue (*Mf*-fruit), however, only *CHI* was up-regulated. The fact that *CHI* expression is induced in both exposures to fruit peel tissues and to fungal mycelia (Figure 8B) may indicate that chitinases are involved in several biological interactions and not specifically in pathogen cell wall degradation.

### Iron homeostasis

Many pathogens utilize secreted siderophores and/or high-affinity uptake systems to acquire iron, and many are able to utilize ferritin, transferrin, lactoferrin, heme and heme-containing proteins for cellular processes related to virulence [29]. Iron competition was reported as the main mode of action of *M. pulcherrima* inhibition of *Botrytis cinerea, Alternaria alternata* and *P. expansum*[24]. Iron is essential for fungal growth and pathogenesis. Iron metabolism has been well characterized in the model yeast *Saccharomyces cerevisiae*. There are two different pathways that allow iron uptake from the environment: the reductive iron-uptake pathway mediated by Fet3p, a multicopper oxidase, and Ftr1p, an iron permease localized on the plasma membrane; and the siderophore-mediated uptake pathway. In our transcriptional analysis, we identified homologs of transmembrane ferric reductases (*FET3*), iron permease (*FTR1*), and an iron transporter (*SIT1*) all of which were up-regulated in the *Mf*-fruit interaction (Additional file 6). This information provides a clue to the potential important role of the reductive iron uptake system in the interaction of *M. fructicola* with fruit tissues. Iron competition was reported as the main mode of action in the ability of *M. pulcherrima* to inhibit *Botrytis cinerea*, *Alternaria alternata* and *P.expansum* infection of apples stored at 1°C for 8 months under controlled atmosphere (2% O_2_ and 3% CO_2_) [24]. *M. fructicola*, similar to *M. pulcherrima*, produces a red pigment that is believed to be associated with the binding of iron (unpublished data). Iron permease (CaFTR1), in *Candida albicans*, was shown to mediate iron acquisition from transferrin and was required for systemic infection [29]. In the plant pathogenic fungus, *Fusarium graminearum,* iron permeases (FgFtr1 and FgFtr2) function in the reductive iron uptake pathway but do not play a major role in pathogenicity [30].

Metal ion homeostasis is interdependent, linking iron availability to that of other metals, like zinc. Zinc itself was reported to be a determinant in cell-cell signaling in *C. albicans* biofilm formation and regulated by the transcription factor, Zap1 [31]. Zap1 has been identified as a regulator of yeast-hypha balance in biofilms through intercellular signaling. Zap1 promotes accumulation of farnesol, an inhibitor of hypha formation in yeasts. Zap1 promotes accumulation of a postulated diffusible yeast cell inhibitor via the control of the zinc transporter, ZTR2. Zap1 activates the expression of the zinc transporter homologs, *ZRT1* and *ZRT2 (z*inc regulated transporter). Overexpression of *ZRT2*, improves the growth of the *zap1*_/*zap1*_ mutant on a low-zinc medium [32].

In our study two transporters, *ZTR3* and *ZTR2*, were up-regulated in the *Mf*-fruit interaction and may be related to low zinc levels on fruit peels. Interestingly, *ZTR2* was down-regulated in cells interacting with fungal mycelia. Zap1 is required for full production of farnesol. Farnesol is believed to be generated from the ergosterol biosynthetic intermediate, farnesyl pyrophosphate [32,33]. *C. albicans* biofilm growth is associated with the overall upregulation of ergosterol biosynthesis as well as increased resistance to antifungal compounds that target ergosterol [34]. Ergosterol biosynthetic genes (ERG) are oppositely regulated by Zap1 in *C. albicans* and *S. cerevisiae*. ERG is positively regulated by ScZAP1 in *S. cerevisiae* and negatively regulated by CaZAP1 in *C. albicans*[32]. The apparently opposite roles of Zap1 in ERG gene regulation in the two organisms may arise from the difference in growth conditions. In our study we observed up-regulation of a group of genes involved in ergosterol metabolism and signaling; *ERG1, ERG 11, ERG5, PSD* and *DAP1* (Additional file 7). Because of the relationship between drug resistance and membrane ergosterol composition, many genes of the ergosterol biosynthetic pathway have been analyzed in *C. albicans*. Pasrija et al. [35] showed that suppressing the expression of an squalene epoxidase *ERG1*, leads to increased sensitivity to a number of drugs, including fluconazole and cycloheximide.

## Conclusions

The results presented in this study provide the first use of RNA-seq, and de novo assembly, to conduct a comprehensive analysis of the transcriptome of *M. fructicola* cells that are in contact with fruit peel tissue or *P. digitatum* mycelia. *De-novo assembly of the Illumina sequences generated a large number of new hypothetical proteins, which were categorized into 43 functional groups, in M. fructicola.* The identification of *M. fructicola* genes associated with biocontrol interactions will form the basis of understanding the biology of biocontrol systems and mechanisms associated with effective biocontrol agents.

## Methods

### Yeast and pathogen cultures

*Metschnikowia fructicola*, Strain 277, [4] was grown in NYDP (nutrient broth (8 g l^-1^), yeast extract (5 g l^-1^), D-glucose (10 gl^-1^) and chloramphenicol (250 mg l^-1^). One ml of the yeast cell suspension was aseptically transferred from 24 h old starter culture to 250 ml. Ehrlenmeyer flasks and place on an orbital shaker at 160 rpm for 24 h at 26°C. Yeast cells were pelleted by centrifugation at 6000 rpm, washed twice with sterile distilled water, re-suspended in sterile water to its initial volume and the cell suspension concentration was adjusted to 1 × 10^8^ cells ml^-1^.

*Penicillium digitatum* was isolated from decayed citrus fruit and maintained on potato dextrose agar (PDA) (Difco, Sparks, MD, USA). Fungal spore suspension was obtained from 2-week-old cultures grown on PDA at 26°C. The number of spores was calculated using a Cellometer (Neubauer, Germany), and the spore concentration was adjusted to 1 × 10^4^ spores ml^-1^.

### Fruit

Red grapefruit ('Star Ruby') were obtained from a local orchard and used within 24 h after harvest. Before use, fruits were washed with tap water and detergent, rinsed, dried and surface disinfected by wiping with technical ethanol (70%).

### *Metschnikowia fructicola* -*Penicillium digitatum* (*Mf-Pdig*) interaction

To obtain an even mycelial mat of *P. digitatum* on PDA, 1 ml of fungal spore suspension (1 × 10^4^ spores ml^-1^) was thoroughly mixed with 9 ml molten 1% PDA (cooled down to about 40°C) in a petri dish and allowed to solidify at room temperature. After 5 days incubation at 25°C, 1 ml of yeast cells in water (1 × 10^8^ cells ml^-1^) was placed on the mycelial mat that formed on the surface of the PDA. Yeast cells were carefully washed several times from the surface of mycelial mats with 1 ml of sterilized distilled water using a pipette after 5, 12, 24 and 48 h of incubation. Washings from 10 plates were collected at each time point, filtered through 4 layers of sterile cheese cloth to remove any mycelia, and transferred into sterile 50 ml tubes. Yeast cells were then pelleted by centrifugation at 6000 rpm, washed twice with sterile distilled water and pelleted again by centrifugation. The pellet was frozen and stored at −80°C for subsequent RNA extraction. Yeast cells gown in NYDB for 5, 12, 24 and 48 h were used as controls following pelleting and washing with water.

### *Metschnikowia fructicola*-fruit (*Mf*-fruit) interaction

*M. fructicola* cells (1 × 10^8^ cell ml^-1^) in water were prepared as indicated above. Surface washed and disinfected grapefruits were peeled using hand peeler to make 3 mm thick strips. Alternatively, discs were cut using a 9 mm diameter cork borer under aseptic conditions. Disks were immersed for 1 min in *M. fructicola* cell suspension (1 × 10^8^ cells ml^-1^) and then transferred into Petri dishes lined with moist, sterile filter paper. After 5, 12, 24 and 48 h of incubation at 25°C under moist conditions, 10 disks from each treatment were transferred to 50 ml sterile conical tubes containing 10 ml sterile water and shaken on an orbital shaker at 180 rpm for 10 min. Fruit disks were then discarded and dislodged yeast cells were collected by centrifugation at 6000 rpm for 10 min, washed twice with sterile distilled water, centrifuged between rinses, and re-suspended in sterile water. Concentration of the cell suspension was adjusted to 1 × 10^7^ cells ml^-1^ and then pelleted by centrifugation. Yeast pellets were frozen and stored at −80°C for subsequent RNA preparation.

### RNA extraction and cDNA synthesis

Total RNA from frozen each yeast pellet was extracted using a MasterPure™ Yeast RNA Purification Kit (Epicentre® Biotechnologies, Madison, WI, USA) accor-ding to the manufacturer`s instructions. Quality and concentration of total RNA was analyzed by gel electrophoresis and a spectrophotometer ND-1000, respectively (NanoDrop, Wilmington, DE, USA). First-strand cDNA was synthesized with a Verso cDNA Kit (Thermo Fisher Scientific, Epson, UK), from 1 μg of total RNA that had been pretreated with 1.5 units of RNase I (Epicentre® Biotechnologies, Madison, WI, USA).

### RNA sequencing

RNA samples isolated from control cells of *M. fructicola* grown in media (*Mf)*, *M. fructicola* – fruit interaction (*Mf*-fruit), and *M. fructicola* – *P. digitatum* (*Mf-Pdig*) after 24 h of incubation were further prepared for RNA sequencing using a MasterPure™ Yeast RNA Purification Kit as described above. cDNA preparation, cDNA library construction, and sequencing were performed at The Genome High-Throughput Sequencing Laboratory, Sackler Faculty of Medicine and George Wise Faculty of Life Sciences, Tel Aviv University. According to the Illumina manufacturer's instructions, poly(A) + RNA was purified from 20 μg of total RNA using oligo(dT) magnetic beads and fragmented into short sequences in the presence of divalent cations at 94°C for 5 min. The cleaved poly(A) + RNA was transcribed and second-strand cDNA synthesis was performed. After end-repair and the ligation of adaptors, cDNA products were amplified by PCR and purified using the QIAquick PCR Purification Kit to create a specific cDNA library. Cluster formation, primer hybridization, and singled-end 76 cycle sequencing were performed on an Illumina Genome Analyzer IIx using proprietary reagents according to the manufacturer’s instructions.

### Bioinformatic analysis of the expression RNA-seq libraries

*De-novo* assembly of the *M. fructicola* transcriptome without a reference genome using the four libraries obtained from the different treatments of the yeast was accomplished using Trinity de-novo transcriptome assembly software [36]. After assembling the *M. fructicola* transcriptome, every RNA-seq library was separately aligned to the generated transcriptome assembly using Bowtie [37]. The counting of alignments was done using RSEM [38]. The differential expression Statistical analysis was done using the statistical method described in the R package DESeq [39]. Annotation of the *de-novo* transcript contigs was done using blast2go software [40]. Using blast2GO software, blastx [41] was performed against the NCBI non-redundant protein database [42] with an E-value cut off of 1e^-5^. Mapped records were associated with matching Gene Ontology (GO) terms in the GO database [43] used the WEGO tool (web gene ontology annotation Plotting) for plotting GO annotation results (http://wego.genomics.org.cn) [44]. Finally, InterProScan [45,46] was used to identify additional GO annotations for our sequences [47]. The transcriptome datasets are available at the NCBI Sequence Read Archive (SRA), under accession number SRA054245.

### Mapping of *Metschnikowia fructicola* unigenes to the draft genome

The Unigene transcript were blasted against the *Metschnikowia fructicola* draft genome available under gene bank accession number ANFW01000000 with cutoff e-value of 1e-50 and identity >85%.

### Phylogenetic tree construction

Sequences of the ribosomal RNA large subunit were downloaded from the SILVA database [48]. Sequences were aligned using MUSCLE program [49]. Then, phylogenetic trees were reconstructed for each dataset based on maximum likelihood (ML) framework using the phyml software with 100 bootstrap replicates [50].

### Real-time quantitative reverse transcriptase PCR

Real-time quantitative reverse transcriptase PCR (RT-qPCR) was performed to validate transcription levels determined by RNA-Seq and to evaluate transcription levels at additional time points (5, 12, 24 and 48 h). A ten μl reaction contained specific primers (200 nM final concentration) Additional file 8, absolute QPCR SYBR Green ROX Mix (ABgene, Epson, UK) and cDNA templates (1:10 dilution), and was performed on a GENE 3000 Real-Time PCR system (Corbett Life Science, Sydney, Australia). The cycle threshold values (CT) were determined and the relative fold differences were calculated by the 2^_ΔΔCT^ method [51] using actin (AJ745127) as the endogenous reference gene [6]. Samples were run in triplicate, and each experiment was repeated twice. Two experiments of independently cultivated yeast were performed to confirm the reproducibility of the results.

## Competing interests

Authors declare that they have no competing interests.

## Authors’ contributions

VH did the experiments and drafted the manuscript. NS did all the bioinformatic analysis and drafted the manuscript. LTS, JL, GR, CK, RA, ML helped with the analysis and experiments or contributed additional analysis. MW and SD conceived the project, supervised the project and drafted the manuscript. All authors have read and approved the final manuscript.

## Supplementary Material

Additional file 1 A fasta file containing all Unigene sequences of *Metchnikowia fructicola* transcriptome. Click here for file

Additional file 2 Annotation of all *Metschnikowia fructicola* unigenes: the gene description, go terms associated and proscan analysis of every transcript. Click here for file

Additional file 3 Mapping of *Metschnikowia fructicola* de-novo assembled transcript to *Metschnikowia fructicola* genome draft genbank accession number ANFW01000000. Click here for file

Additional file 4 The differentially expressed genes in *Metschnikowia fructicola* interaction with *Penicilium digitatum* (*Mf-Pdig*) and interaction with fruit (*Mf*-fruit) and with fruit (*p* < 0.05). Click here for file

Additional file 5 Summary of differential expressed genes in *Metschnikowia fructicola* interaction with *Penicillium digitatum* and interaction with fruit (*p* < 0.05) involved in the response to stresses (chemical GO:042221), oxidative (GO:006979), osmotic (GO:006970), heat (GO:009408), starvation (GO:042594), DNA damage stimulus (GO:006974). Click here for file

Additional file 6 Summary of differential expressed genes in *Metschnikowia fructicola* interaction with *Penicillium digitatum* and interaction with fruit (*p* < 0.05) involved in transport (transmembrane GO:055085), ion (GO:006811), carbohydrate (GO:008643), lipid (GO:006869), amino acid transport (GO:006865). Click here for file

Additional file 7 Summary of differential expressed genes in *Metschnikowia fructicola* interaction with *Penicillium digitatum* and interaction with fruit (p < 0.05) involved in metabolic (lipid GO:006629), (vitamin GO:006766), (cofactor GO:051186), (cellular amino acid GO:006520) processes. Click here for file

Additional file 8 The primer sequences used in qPCR. Click here for file
