# Supplementary material for: De-novo assembly and characterization of the transcriptome of Metschnikowia fructicola reveals differences in gene expression following interaction with Penicillium digitatum and grapefruit peel
Source: BMC Genomics. 2013 Mar 12;14:168. doi: 10.1186/1471-2164-14-168 (PMC3608080; doi:10.1186/1471-2164-14-168)
Supplement: Additional file 4 — The differentially expressed genes in Metschnikowia fructicola interaction with Penicilium digitatum (Mf-Pdig) and interaction with fruit (Mf-fruit) and with fruit (p < 0.05). [file 1471-2164-14-168-S4.docx]

***De-novo* assembly and characterization of the transcriptome of *Metschnikowia fructicola* reveals differences in gene expression following interaction with *Penicillium digitatum* and grapefruit peel**

**Vera Hershkovitz^1,^** **^†^**

Email: vhershko@agri.gov.il

**Noa Sela^2, †^**

Email: [noa@agri.gov.il](mailto:noa@agri.gov.il)

**Leena Taha-Salaime^1,3,4^**

Email: [leena.taha@mail.huji.ac.il](mailto:leena.taha@mail.huji.ac.il)

**Jia Liu^5^**

Email:Jia.Liu@ARS.USDA.GOV

**Ginat Rafael^1^**

Email: [pongie@volcani.agri.gov.il](mailto:pongie@volcani.agri.gov.il)

**Clarita Kessler^1^**

Email: [clarita.bendayan@gmail.com](mailto:clarita.bendayan@gmail.com)

**Radi Aly^3^**

Email: [radi@volcani.agri.gov.il](mailto:radi@volcani.agri.gov.il)

**Maggie Levy^4^**

Email: [levym@agri.huji.ac.il](mailto:levym@agri.huji.ac.il)

**Michael Wisniewski^5^**

Email: Michael.Wisniewski@ARS.USDA.GOV

**Samir Droby^1*^**

* Corresponding author

Email: samird[@volcani.agri.gov.il](mailto:samird@volcani.agri.gov.il)

**^1^** Department of Postharvest and Food Sciences, ARO, the Volcani Center, Bet Dagan 50250, Israel

^2^ Department of Plant Pathology and Weed Research, ARO, the Volcani Center, Bet Dagan 50250, Israel

^3^ Department of Plant Pathology and Weed Research, the Volcani Center, Newe-Yaar Research Center, Israel.

^4^ [Department of Plant Pathology and Microbiology](http://departments.agri.huji.ac.il/plantpath/), [the Robert H. Smith Faculty of Agriculture, Food and Environment ,](http://www.agri.huji.ac.il/) [the Hebrew University of Jerusalem](http://www.huji.ac.il/), Israel.

^5^ U.S. Department of Agriculture-Agricultural Research Service (USDA-ARS), Appalachian Fruit Research Station, WV, USA.

† Equal contributors.

**Table S2**. Summary of differential expressed genes in *M. fructicola* interaction with *P. digitatum* (*Mf-Pdig*) and interaction with fruit (*Mf*-fruit) and with fruit ( *p* < 0.05)

| id | SC matching gene | log2FoldChange | pval | description |
| --- | --- | --- | --- | --- |
| *Mf-Pdig* interaction | | | | |
| comp6540_c0 | YAL025C | 2.50 | 0.05 | Essential nuclear protein, constituent of 66S pre- ribosomal particles; required for maturation of 25S and 5.8S rRNAs; required for maintenance of M1 satellite double-stranded RNA of the L-A virus [Source:SGD;Acc:S000000023] |
| comp1736_c0 | YAL038W | 2.64 | 0.04 | Pyruvate kinase, functions as a homotetramer in glycolysis to convert phosphoenolpyruvate to pyruvate, the input for aerobic (TCA cycle) or anaerobic (glucose fermentation) respiration [Source:SGD;Acc:S000000036] |
| comp458_c0 | YBL092W | 2.61 | 0.04 | Protein component of the large (60S) ribosomal subunit, has similarity to rat L32 ribosomal protein; overexpression disrupts telomeric silencing [Source:SGD;Acc:S000000188] |
| comp10394_c0 | YBR093C | 3.13 | 0.03 | Repressible acid phosphatase (1 of 3) that also mediates extracellular nucleotide-derived phosphate hydrolysis; secretory pathway derived cell surface glycoprotein; induced by phosphate starvation and coordinately regulated by PHO4 and PHO2 [Source:SGD;Acc:S000000297] |
| comp1753_c0 | YBR094W | 3.22 | 0.01 | Putative tubulin tyrosine ligase associated with P- bodies [Source:SGD;Acc:S000000298] |
| comp9619_c0 | YBR102C | -4.36 | 0.05 | Essential protein with dual roles in spliceosome assembly and exocytosis; the exocyst complex (Sec3p, Sec5p, Sec6p, Sec8p, Sec10p, Sec15p, Exo70p, and Exo84p) mediates polarized targeting of secretory vesicles to active sites of exocytosis [Source:SGD;Acc:S000000306] |
| comp615_c0 | YBR189W | 2.65 | 0.04 | Protein component of the small (40S) ribosomal subunit; nearly identical to Rps9Ap and has similarity to E. coli S4 and rat S9 ribosomal proteins [Source:SGD;Acc:S000000393] |
| comp490_c0 | YBR191W | 2.51 | 0.05 | Protein component of the large (60S) ribosomal subunit, nearly identical to Rpl21Bp and has similarity to rat L21 ribosomal protein [Source:SGD;Acc:S000000395] |
| comp4427_c0 | YCL069W | 2.54 | 0.05 | Permease of basic amino acids in the vacuolar membrane [Source:SGD;Acc:S000000574] |
| comp2258_c0 | YCR019W | -2.51 | 0.05 | Protein necessary for structural stability of L-A double-stranded RNA-containing particles [Source:SGD;Acc:S000000612] |
| comp656_c0 | YCR031C | 2.45 | 0.05 | Ribosomal protein 59 of the small subunit, required for ribosome assembly and 20S pre-rRNA processing; mutations confer cryptopleurine resistance; nearly identical to Rps14Bp and similar to E. coli S11 and rat S14 ribosomal proteins [Source:SGD;Acc:S000000627] |
| comp8775_c0 | YCR098C | 3.89 | 0.01 | Plasma membrane permease, mediates uptake of glycerophosphoinositol and glycerophosphocholine as sources of the nutrients inositol and phosphate; expression and transport rate are regulated by phosphate and inositol availability [Source:SGD;Acc:S000000695] |
| comp2840_c0 | YCR098C | 2.50 | 0.05 | Plasma membrane permease, mediates uptake of glycerophosphoinositol and glycerophosphocholine as sources of the nutrients inositol and phosphate; expression and transport rate are regulated by phosphate and inositol availability [Source:SGD;Acc:S000000695] |
| comp604_c0 | YDL075W | 2.48 | 0.05 | Protein component of the large (60S) ribosomal subunit, nearly identical to Rpl31Bp and has similarity to rat L31 ribosomal protein; associates with the karyopherin Sxm1p; loss of both Rpl31p and Rpl39p confers lethality [Source:SGD;Acc:S000002233] |
| comp473_c0 | YDL083C | 2.56 | 0.04 | Protein component of the small (40S) ribosomal subunit; identical to Rps16Ap and has similarity to E. coli S9 and rat S16 ribosomal proteins [Source:SGD;Acc:S000002241] |
| comp462_c0 | YDL191W | 2.66 | 0.04 | Protein component of the large (60S) ribosomal subunit, identical to Rpl35Bp and has similarity to rat L35 ribosomal protein [Source:SGD;Acc:S000002350] |
| comp928_c0 | YDL194W | 4.28 | 0.00 | Plasma membrane low glucose sensor that regulates glucose transport; contains 12 predicted transmembrane segments and a long C-terminal tail required for induction of hexose transporters; also senses fructose and mannose; similar to Rgt2p [Source:SGD;Acc:S000002353] |
| comp2545_c0 | YDL222C | 2.52 | 0.05 | Integral membrane protein localized to mitochondria (untagged protein); required for sporulation and maintaining sphingolipid content; has sequence similarity to SUR7 and YNL194C [Source:SGD;Acc:S000002381] |
| comp6782_c0 | YDR011W | 2.68 | 0.04 | Plasma membrane ATP-binding cassette (ABC) transporter, multidrug transporter involved in multidrug resistance and resistance to singlet oxygen species [Source:SGD;Acc:S000002418] |
| comp5856_c0 | YDR011W | 2.55 | 0.05 | Plasma membrane ATP-binding cassette (ABC) transporter, multidrug transporter involved in multidrug resistance and resistance to singlet oxygen species [Source:SGD;Acc:S000002418] |
| comp666_c0 | YDR025W | 2.50 | 0.05 | Protein component of the small (40S) ribosomal subunit; identical to Rps11Bp and has similarity to E. coli S17 and rat S11 ribosomal proteins [Source:SGD;Acc:S000002432] |
| comp134_c0 | YDR214W | -2.43 | 0.05 | Co-chaperone that binds to Hsp82p and activates its ATPase activity; similar to Hch1p; expression is regulated by stresses such as heat shock [Source:SGD;Acc:S000002622] |
| comp39_c0 | YDR258C | -2.44 | 0.05 | Oligomeric mitochondrial matrix chaperone that cooperates with Ssc1p in mitochondrial thermotolerance after heat shock; able to prevent the aggregation of misfolded proteins as well as resolubilize protein aggregates [Source:SGD;Acc:S000002666] |
| comp2682_c0 | YDR343C | 2.54 | 0.05 | High-affinity glucose transporter of the major facilitator superfamily, nearly identical to Hxt7p, expressed at high basal levels relative to other HXTs, repression of expression by high glucose requires SNF3 [Source:SGD;Acc:S000002751] |
| comp486_c0 | YDR447C | 2.47 | 0.05 | Ribosomal protein 51 (rp51) of the small (40s) subunit; nearly identical to Rps17Ap and has similarity to rat S17 ribosomal protein [Source:SGD;Acc:S000002855] |
| comp538_c0 | YDR500C | 2.73 | 0.03 | Protein component of the large (60S) ribosomal subunit, has similarity to Rpl37Ap and to rat L37 ribosomal protein [Source:SGD;Acc:S000002908] |
| comp334_c0 | YDR536W | -2.52 | 0.05 | Glycerol proton symporter of the plasma membrane, subject to glucose-induced inactivation, strongly but transiently induced when cells are subjected to osmotic shock [Source:SGD;Acc:S000002944] |
| comp401_c0 | YEL054C | 2.63 | 0.04 | Protein component of the large (60S) ribosomal subunit, nearly identical to Rpl12Bp; rpl12a rpl12b double mutant exhibits slow growth and slow translation; has similarity to E. coli L11 and rat L12 ribosomal proteins [Source:SGD;Acc:S000000780] |
| comp1249_c0 | YEL063C | 2.74 | 0.03 | Plasma membrane arginine permease, requires phosphatidyl ethanolamine (PE) for localization, exclusively associated with lipid rafts; mutation confers canavanine resistance [Source:SGD;Acc:S000000789] |
| comp2610_c0 | YER019W | -2.54 | 0.05 | Mitochondrial membrane localized inositol phosphosphingolipid phospholipase C, hydrolyzes complex sphingolipids to produce ceramide; activated by phosphatidylserine, cardiolipin, and phosphatidylglycerol; mediates Na+ and Li+ halotolerance [Source:SGD;Acc:S000000821] |
| comp464_c0 | YER131W | 2.59 | 0.04 | Protein component of the small (40S) ribosomal subunit; nearly identical to Rps26Ap and has similarity to rat S26 ribosomal protein [Source:SGD;Acc:S000000933] |
| comp5793_c0 | YFL055W | 2.51 | 0.05 | Low-affinity amino acid permease, may act to supply the cell with amino acids as nitrogen source in nitrogen- poor conditions; transcription is induced under conditions of sulfur limitation; plays a role in regulating Ty1 transposition [Source:SGD;Acc:S000001839] |
| comp721_c0 | YFR032C-A | 2.46 | 0.05 | Protein component of the large (60S) ribosomal subunit, has similarity to rat L29 ribosomal protein; not essential for translation, but required for proper joining of the large and small ribosomal subunits and for normal translation rate [Source:SGD;Acc:S000006437] |
| comp1005_c0 | YGL006W | 2.68 | 0.03 | Vacuolar Ca2+ ATPase involved in depleting cytosol of Ca2+ ions; prevents growth inhibition by activation of calcineurin in the presence of elevated concentrations of calcium; similar to mammalian PMCA1a [Source:SGD;Acc:S000002974] |
| comp537_c0 | YGL030W | 2.76 | 0.03 | Protein component of the large (60S) ribosomal subunit, has similarity to rat L30 ribosomal protein; involved in pre-rRNA processing in the nucleolus; autoregulates splicing of its transcript [Source:SGD;Acc:S000002998] |
| comp683_c0 | YGL103W | 2.53 | 0.05 | Ribosomal protein of the large (60S) ribosomal subunit, has similarity to E. coli L15 and rat L27a ribosomal proteins; may have peptidyl transferase activity; can mutate to cycloheximide resistance [Source:SGD;Acc:S000003071] |
| comp1251_c0 | YGR019W | 3.33 | 0.01 | Gamma-aminobutyrate (GABA) transaminase (4- aminobutyrate aminotransferase) involved in the 4- aminobutyrate and glutamate degradation pathways; required for normal oxidative stress tolerance and nitrogen utilization [Source:SGD;Acc:S000003251] |
| comp689_c0 | YGR034W | 2.68 | 0.03 | Protein component of the large (60S) ribosomal subunit, nearly identical to Rpl26Ap and has similarity to E. coli L24 and rat L26 ribosomal proteins; binds to 5.8S rRNA [Source:SGD;Acc:S000003266] |
| comp597_c0 | YGR085C | 2.45 | 0.05 | Protein component of the large (60S) ribosomal subunit, nearly identical to Rpl11Ap; involved in ribosomal assembly; depletion causes degradation of proteins and RNA of the 60S subunit; has similarity to E. coli L5 and rat L11 [Source:SGD;Acc:S000003317] |
| comp2447_c0 | YGR281W | 2.45 | 0.05 | Plasma membrane ATP-binding cassette (ABC) transporter, multidrug transporter mediates export of many different organic anions including oligomycin; similar to human cystic fibrosis transmembrane receptor (CFTR) [Source:SGD;Acc:S000003513] |
| comp523_c0 | YHL001W | 2.44 | 0.05 | Protein component of the large (60S) ribosomal subunit, nearly identical to Rpl14Ap and has similarity to rat L14 ribosomal protein [Source:SGD;Acc:S000000993] |
| comp113_c0 | YHR006W | -3.09 | 0.02 | Transcription factor, activated by proteolytic processing in response to signals from the SPS sensor system for external amino acids; activates transcription of amino acid permease genes [Source:SGD;Acc:S000001048] |
| comp1119_c0 | YHR021C | 2.76 | 0.03 | Protein component of the small (40S) ribosomal subunit; nearly identical to Rps27Ap and has similarity to rat S27 ribosomal protein [Source:SGD;Acc:S000001063] |
| comp6236_c0 | YHR048W | 3.38 | 0.01 | Presumed antiporter of the DHA1 family of multidrug resistance transporters; contains 12 predicted transmembrane spans; expression of gene is up-regulated in cells exhibiting reduced susceptibility to azoles [Source:SGD;Acc:S000001090] |
| comp5468_c0 | YHR092C | 2.72 | 0.04 | High-affinity glucose transporter of the major facilitator superfamily, expression is induced by low levels of glucose and repressed by high levels of glucose [Source:SGD;Acc:S000001134] |
| comp5455_c0 | YHR188C | -4.86 | 0.02 | Transmembrane protein subunit of the glycosylphosphatidylinositol transamidase complex that adds GPIs to newly synthesized proteins; human PIG-Tp homolog [Source:SGD;Acc:S000001231] |
| comp552_c0 | YIL052C | 2.46 | 0.05 | Protein component of the large (60S) ribosomal subunit, nearly identical to Rpl34Ap and has similarity to rat L34 ribosomal protein [Source:SGD;Acc:S000001314] |
| comp499_c0 | YIL053W | 2.40 | 0.06 | Constitutively expressed isoform of DL-glycerol-3- phosphatase; involved in glycerol biosynthesis, induced in response to both anaerobic and, along with the Hor2p/Gpp2p isoform, osmotic stress [Source:SGD;Acc:S000001315] |
| comp7242_c0 | YJL153C | 4.47 | 0.00 | Inositol-3-phosphate synthase, involved in synthesis of inositol phosphates and inositol-containing phospholipids; transcription is coregulated with other phospholipid biosynthetic genes by Ino2p and Ino4p, which bind the UASINO DNA element [Source:SGD;Acc:S000003689] |
| comp9040_c0 | YJL153C | 4.21 | 0.00 | Inositol-3-phosphate synthase, involved in synthesis of inositol phosphates and inositol-containing phospholipids; transcription is coregulated with other phospholipid biosynthetic genes by Ino2p and Ino4p, which bind the UASINO DNA element [Source:SGD;Acc:S000003689] |
| comp7235_c0 | YJL153C | 3.18 | 0.02 | Inositol-3-phosphate synthase, involved in synthesis of inositol phosphates and inositol-containing phospholipids; transcription is coregulated with other phospholipid biosynthetic genes by Ino2p and Ino4p, which bind the UASINO DNA element [Source:SGD;Acc:S000003689] |
| comp632_c0 | YJL189W | 2.91 | 0.02 | Protein component of the large (60S) ribosomal subunit, has similarity to rat L39 ribosomal protein; required for ribosome biogenesis; loss of both Rpl31p and Rpl39p confers lethality; also exhibits genetic interactions with SIS1 and PAB1 [Source:SGD;Acc:S000003725] |
| comp884_c0 | YJL190C | 2.42 | 0.05 | Protein component of the small (40S) ribosomal subunit; nearly identical to Rps22Bp and has similarity to E. coli S8 and rat S15a ribosomal proteins [Source:SGD;Acc:S000003726] |
| comp513_c0 | YJR104C | -2.98 | 0.02 | Cytosolic copper-zinc superoxide dismutase; some mutations are analogous to those that cause ALS (amyotrophic lateral sclerosis) in humans [Source:SGD;Acc:S000003865] |
| comp9071_c0 | YJR109C | 3.42 | 0.01 | Large subunit of carbamoyl phosphate synthetase, which catalyzes a step in the synthesis of citrulline, an arginine precursor [Source:SGD;Acc:S000003870] |
| comp4738_c0 | YJR109C | 3.09 | 0.02 | Large subunit of carbamoyl phosphate synthetase, which catalyzes a step in the synthesis of citrulline, an arginine precursor [Source:SGD;Acc:S000003870] |
| comp620_c0 | YJR123W | 2.60 | 0.04 | Protein component of the small (40S) ribosomal subunit, the least basic of the non-acidic ribosomal proteins; phosphorylated in vivo; essential for viability; has similarity to E. coli S7 and rat S5 ribosomal proteins [Source:SGD;Acc:S000003884] |
| comp1015_c0 | YJR156C | -2.80 | 0.03 | Protein involved in synthesis of the thiamine precursor hydroxymethylpyrimidine (HMP); member of a subtelomeric gene family including THI5, THI11, THI12, and THI13 [Source:SGD;Acc:S000003917] |
| comp10104_c0 | YKL029C | 3.32 | 0.02 | Mitochondrial malic enzyme, catalyzes the oxidative decarboxylation of malate to pyruvate, which is a key intermediate in sugar metabolism and a precursor for synthesis of several amino acids [Source:SGD;Acc:S000001512] |
| comp7271_c0 | YKL029C | 3.11 | 0.02 | Mitochondrial malic enzyme, catalyzes the oxidative decarboxylation of malate to pyruvate, which is a key intermediate in sugar metabolism and a precursor for synthesis of several amino acids [Source:SGD;Acc:S000001512] |
| comp3793_c0 | YKL029C | 3.08 | 0.02 | Mitochondrial malic enzyme, catalyzes the oxidative decarboxylation of malate to pyruvate, which is a key intermediate in sugar metabolism and a precursor for synthesis of several amino acids [Source:SGD;Acc:S000001512] |
| comp4457_c0 | YKL029C | 2.79 | 0.03 | Mitochondrial malic enzyme, catalyzes the oxidative decarboxylation of malate to pyruvate, which is a key intermediate in sugar metabolism and a precursor for synthesis of several amino acids [Source:SGD;Acc:S000001512] |
| comp109_c0 | YKL096W | -2.47 | 0.05 | Cell wall mannoprotein that localizes specifically to birth scars of daughter cells, linked to a beta-1,3- and beta-1,6-glucan heteropolymer through a phosphodiester bond; required for propionic acid resistance [Source:SGD;Acc:S000001579] |
| comp430_c0 | YKL180W | 2.41 | 0.06 | Protein component of the large (60S) ribosomal subunit, nearly identical to Rpl17Bp and has similarity to E. coli L22 and rat L17 ribosomal proteins; copurifies with the Dam1 complex (aka DASH complex) [Source:SGD;Acc:S000001663] |
| comp1140_c0 | YKR039W | 4.28 | 0.00 | General amino acid permease; Gap1p senses the presence of amino acid substrates to regulate localization to the plasma membrane when needed [Source:SGD;Acc:S000001747] |
| comp543_c0 | YKR057W | 2.42 | 0.05 | Protein component of the small (40S) ribosomal subunit; nearly identical to Rps21Bp and has similarity to rat S21 ribosomal protein [Source:SGD;Acc:S000001765] |
| comp3135_c0 | YKR105C | 3.05 | 0.02 | Putative transporter of the Major Facilitator Superfamily (MFS); proposed role as a basic amino acid permease based on phylogeny [Source:SGD;Acc:S000001813] |
| comp2555_c0 | YLL028W | 3.66 | 0.01 | Polyamine transporter that recognizes spermine, putrescine, and spermidine; catalyzes uptake of polyamines at alkaline pH and excretion at acidic pH; phosphorylation enhances activity and sorting to the plasma membrane [Source:SGD;Acc:S000003951] |
| comp7086_c0 | YLL028W | 2.94 | 0.03 | Polyamine transporter that recognizes spermine, putrescine, and spermidine; catalyzes uptake of polyamines at alkaline pH and excretion at acidic pH; phosphorylation enhances activity and sorting to the plasma membrane [Source:SGD;Acc:S000003951] |
| comp4588_c0 | YLL028W | 2.62 | 0.05 | Polyamine transporter that recognizes spermine, putrescine, and spermidine; catalyzes uptake of polyamines at alkaline pH and excretion at acidic pH; phosphorylation enhances activity and sorting to the plasma membrane [Source:SGD;Acc:S000003951] |
| comp220_c0 | YLL039C | 2.58 | 0.04 | Ubiquitin, becomes conjugated to proteins, marking them for selective degradation via the ubiquitin-26S proteasome system; essential for the cellular stress response; encoded as a polyubiquitin precursor comprised of 5 head-to-tail repeats [Source:SGD;Acc:S000003962] |
| comp675_c0 | YLR061W | 2.84 | 0.03 | Protein component of the large (60S) ribosomal subunit, has similarity to Rpl22Bp and to rat L22 ribosomal protein [Source:SGD;Acc:S000004051] |
| comp10007_c0 | YLR106C | 2.79 | 0.04 | Huge dynein-related AAA-type ATPase (midasin), forms extended pre-60S particle with the Rix1 complex (Rix1p-Ipi1p-Ipi3p); acts in removal of ribosomal biogenesis factors at successive steps of pre-60S assembly and export from nucleus [Source:SGD;Acc:S000004096] |
| comp2025_c0 | YLR130C | -2.80 | 0.03 | Low-affinity zinc transporter of the plasma membrane; transcription is induced under low-zinc conditions by the Zap1p transcription factor [Source:SGD;Acc:S000004120] |
| comp3052_c0 | YLR130C | -2.87 | 0.04 | Low-affinity zinc transporter of the plasma membrane; transcription is induced under low-zinc conditions by the Zap1p transcription factor [Source:SGD;Acc:S000004120] |
| comp189_c0 | YLR214W | -5.39 | 0.00 | Ferric reductase and cupric reductase, reduces siderophore-bound iron and oxidized copper prior to uptake by transporters; expression induced by low copper and iron levels [Source:SGD;Acc:S000004204] |
| comp9902_c0 | YLR309C | #NAME? | 0.03 | Protein involved in vesicular transport, mediates transport between an endosomal compartment and the Golgi, contains a Golgi-localization (GRIP) domain that interacts with activated Arl1p-GTP to localize Imh1p to the Golgi [Source:SGD;Acc:S000004300] |
| comp784_c0 | YLR325C | 2.75 | 0.03 | Protein component of the large (60S) ribosomal subunit, has similarity to rat L38 ribosomal protein [Source:SGD;Acc:S000004317] |
| comp399_c0 | YLR333C | 2.51 | 0.05 | Protein component of the small (40S) ribosomal subunit; nearly identical to Rps25Ap and has similarity to rat S25 ribosomal protein [Source:SGD;Acc:S000004325] |
| comp2472_c0 | YLR367W | 2.63 | 0.04 | Protein component of the small (40S) ribosomal subunit; nearly identical to Rps22Ap and has similarity to E. coli S8 and rat S15a ribosomal proteins [Source:SGD;Acc:S000004359] |
| comp720_c0 | YLR388W | 2.84 | 0.03 | Protein component of the small (40S) ribosomal subunit; nearly identical to Rps29Bp and has similarity to rat S29 and E. coli S14 ribosomal proteins [Source:SGD;Acc:S000004380] |
| comp5796_c0 | YLR438W | 2.74 | 0.04 | L-ornithine transaminase (OTAse), catalyzes the second step of arginine degradation, expression is dually- regulated by allophanate induction and a specific arginine induction process; not nitrogen catabolite repression sensitive [Source:SGD;Acc:S000004430] |
| comp368_c0 | YML063W | 2.43 | 0.05 | Ribosomal protein 10 (rp10) of the small (40S) subunit; nearly identical to Rps1Ap and has similarity to rat S3a ribosomal protein [Source:SGD;Acc:S000004528] |
| comp1973_c0 | YML119W | 2.97 | 0.02 | Putative protein of unknown function; YML119W is not an essential gene; potential Cdc28p substrate [Source:SGD;Acc:S000004588] |
| comp1288_c0 | YML123C | 3.37 | 0.01 | High-affinity inorganic phosphate (Pi) transporter and low-affinity manganese transporter; regulated by Pho4p and Spt7p; mutation confers resistance to arsenate; exit from the ER during maturation requires Pho86p [Source:SGD;Acc:S000004592] |
| comp9998_c0 | YMR020W | 3.53 | 0.01 | Polyamine oxidase, converts spermine to spermidine, which is required for the essential hypusination modification of translation factor eIF-5A; also involved in pantothenic acid biosynthesis [Source:SGD;Acc:S000004622] |
| comp5723_c0 | YMR020W | 2.99 | 0.02 | Polyamine oxidase, converts spermine to spermidine, which is required for the essential hypusination modification of translation factor eIF-5A; also involved in pantothenic acid biosynthesis [Source:SGD;Acc:S000004622] |
| comp1311_c0 | YMR086W | -2.46 | 0.05 | Component of the eisosome with unknown function; may interact with ribosomes, based on co-purification experiments; GFP-fusion protein localizes to the cell periphery; expression is repressed by cAMP [Source:SGD;Acc:S000004692] |
| comp292_c0 | YMR121C | 2.44 | 0.05 | Protein component of the large (60S) ribosomal subunit, nearly identical to Rpl15Ap and has similarity to rat L15 ribosomal protein; binds to 5.8 S rRNA [Source:SGD;Acc:S000004728] |
| comp1223_c0 | YMR240C | -4.25 | 0.00 | Protein required for assembly of U2 snRNP into the spliceosome, forms a complex with Hsh49p and Hsh155p [Source:SGD;Acc:S000004853] |
| comp31_c0 | YMR303C | -3.15 | 0.01 | Glucose-repressible alcohol dehydrogenase II, catalyzes the conversion of ethanol to acetaldehyde; involved in the production of certain carboxylate esters; regulated by ADR1 [Source:SGD;Acc:S000004918] |
| comp628_c0 | YNL067W | 2.73 | 0.03 | Protein component of the large (60S) ribosomal subunit, nearly identical to Rpl9Ap and has similarity to E. coli L6 and rat L9 ribosomal proteins [Source:SGD;Acc:S000005011] |
| comp325_c0 | YNL069C | 2.55 | 0.04 | N-terminally acetylated protein component of the large (60S) ribosomal subunit, binds to 5.8 S rRNA; has similarity to Rpl16Ap, E. coli L13 and rat L13a ribosomal proteins; transcriptionally regulated by Rap1p [Source:SGD;Acc:S000005013] |
| comp8962_c0 | YNL142W | 3.08 | 0.02 | Ammonium permease involved in regulation of pseudohyphal growth; belongs to a ubiquitous family of cytoplasmic membrane proteins that transport only ammonium (NH4+); expression is under the nitrogen catabolite repression regulation [Source:SGD;Acc:S000005086] |
| comp705_c0 | YNL162W | 2.54 | 0.04 | Protein component of the large (60S) ribosomal subunit, identical to Rpl42Bp and has similarity to rat L44 ribosomal protein [Source:SGD;Acc:S000005106] |
| comp585_c0 | YNL301C | 2.46 | 0.05 | Protein component of the large (60S) ribosomal subunit, identical to Rpl18Ap and has similarity to rat L18 ribosomal protein [Source:SGD;Acc:S000005245] |
| comp6626_c0 | YNR070W | 2.85 | 0.03 | Putative transporter of the ATP-binding cassette (ABC) family, implicated in pleiotropic drug resistance; the authentic, non-tagged protein is detected in highly purified mitochondria in high-throughput studies [Source:SGD;Acc:S000005353] |
| comp2881_c0 | YOL058W | 2.61 | 0.04 | Arginosuccinate synthetase, catalyzes the formation of L-argininosuccinate from citrulline and L-aspartate in the arginine biosynthesis pathway; potential Cdc28p substrate [Source:SGD;Acc:S000005419] |
| comp201_c0 | YOL103W | 2.81 | 0.03 | Myo-inositol transporter with strong similarity to the major myo-inositol transporter Itr1p, member of the sugar transporter superfamily; expressed constitutively [Source:SGD;Acc:S000005463] |
| comp578_c0 | YOL127W | 2.78 | 0.03 | Primary rRNA-binding ribosomal protein component of the large (60S) ribosomal subunit, has similarity to E. coli L23 and rat L23a ribosomal proteins; binds to 25S rRNA via a conserved C-terminal motif [Source:SGD;Acc:S000005487] |
| comp283_c0 | YOL155C | -3.15 | 0.01 | Haze-protective mannoprotein that reduces the particle size of aggregated proteins in white wines [Source:SGD;Acc:S000005515] |
| comp1364_c0 | YOR049C | 3.10 | 0.02 | Suppressor of sphingoid long chain base (LCB) sensitivity of an LCB-lyase mutation; putative integral membrane transporter or flippase that may transport LCBs from the cytoplasmic side toward the extracytoplasmic side of the membrane [Source:SGD;Acc:S000005575] |
| comp413_c0 | YOR234C | 2.63 | 0.04 | Ribosomal protein L37 of the large (60S) ribosomal subunit, nearly identical to Rpl33Ap and has similarity to rat L35a; rpl33b null mutant exhibits normal growth while rpl33a rpl33b double null mutant is inviable [Source:SGD;Acc:S000005760] |
| comp474_c0 | YOR293W | 2.55 | 0.04 | Protein component of the small (40S) ribosomal subunit; nearly identical to Rps10Bp and has similarity to rat ribosomal protein S10 [Source:SGD;Acc:S000005819] |
| comp544_c0 | YOR312C | 2.80 | 0.03 | Protein component of the large (60S) ribosomal subunit, nearly identical to Rpl20Ap and has similarity to rat L18a ribosomal protein [Source:SGD;Acc:S000005839] |
| comp979_c0 | YOR369C | 2.84 | 0.03 | Protein component of the small (40S) ribosomal subunit; has similarity to rat ribosomal protein S12 [Source:SGD;Acc:S000005896] |
| comp947_c0 | YOR374W | 2.71 | 0.03 | Mitochondrial aldehyde dehydrogenase, required for growth on ethanol and conversion of acetaldehyde to acetate; phosphorylated; activity is K+ dependent; utilizes NADP+ or NAD+ equally as coenzymes; expression is glucose repressed [Source:SGD;Acc:S000005901] |
| comp661_c1 | YPL179W | 2.85 | 0.03 | Putative protein serine/threonine phosphatase; null mutation enhances efficiency of translational suppressors [Source:SGD;Acc:S000006100] |
| comp4014_c0 | YPL226W | 2.81 | 0.03 | ATP binding cassette protein that cosediments with polysomes and is required for biogenesis of the small ribosomal subunit; Asn/Gln-rich rich region supports [NU+] prion formation and susceptibility to [PSI+] prion induction [Source:SGD;Acc:S000006147] |
| comp7634_c0 | YPR055W | -4.98 | 0.02 | Essential 121kDa subunit of the exocyst complex (Sec3p, Sec5p, Sec6p, Sec8p, Sec10p, Sec15p, Exo70p, and Exo84p), which has the essential function of mediating polarized targeting of secretory vesicles to active sites of exocytosis [Source:SGD;Acc:S000006259] |
| comp5533_c0 | YPR194C | 3.23 | 0.01 | Oligopeptide transporter; member of the OPT family, with potential orthologs in S. pombe and C. albicans; also plays a role in formation of mature vacuoles [Source:SGD;Acc:S000006398] |
| comp163_c0 | YBR126C | -3.81 | 0.05 | Synthase subunit of trehalose-6-phosphate synthase/phosphatase complex, which synthesizes the storage carbohydrate trehalose; also found in a monomeric form; expression is induced by the stress response and repressed by the Ras-cAMP pathway [Source:SGD;Acc:S000000330] |
| comp2840_c0 | YCR098C | 3.94 | 0.05 | Plasma membrane permease, mediates uptake of glycerophosphoinositol and glycerophosphocholine as sources of the nutrients inositol and phosphate; expression and transport rate are regulated by phosphate and inositol availability [Source:SGD;Acc:S000000695] |
| comp928_c0 | YDL194W | 4.06 | 0.04 | Plasma membrane low glucose sensor that regulates glucose transport; contains 12 predicted transmembrane segments and a long C-terminal tail required for induction of hexose transporters; also senses fructose and mannose; similar to Rgt2p [Source:SGD;Acc:S000002353] |
| comp3071_c0 | YDR150W | -4.15 | 0.05 | Protein required for nuclear migration, localizes to the mother cell cortex and the bud tip; may mediate interactions of dynein and cytoplasmic microtubules with the cell cortex [Source:SGD;Acc:S000002557] |
| comp32_c0 | YDR251W | -7.79 | 0.00 | Essential protein of unknown function; exhibits variable expression during colony morphogenesis; overexpression permits survival without protein phosphatase 2A, inhibits growth, and induces a filamentous phenotype [Source:SGD;Acc:S000002659] |
| comp6779_c0 | YDR345C | 5.02 | 0.02 | Low affinity glucose transporter of the major facilitator superfamily, expression is induced in low or high glucose conditions [Source:SGD;Acc:S000002753] |
| comp334_c0 | YDR536W | -4.79 | 0.02 | Glycerol proton symporter of the plasma membrane, subject to glucose-induced inactivation, strongly but transiently induced when cells are subjected to osmotic shock [Source:SGD;Acc:S000002944] |
| comp1249_c0 | YEL063C | 4.48 | 0.03 | Plasma membrane arginine permease, requires phosphatidyl ethanolamine (PE) for localization, exclusively associated with lipid rafts; mutation confers canavanine resistance [Source:SGD;Acc:S000000789] |
| comp7737_c0 | YEL065W | 3.92 | 0.05 | Ferrioxamine B transporter, member of the ARN family of transporters that specifically recognize siderophore-iron chelates; transcription is induced during iron deprivation and diauxic shift; potentially phosphorylated by Cdc28p [Source:SGD;Acc:S000000791] |
| comp2116_c0 | YGR092W | -4.26 | 0.04 | Ser/Thr kinase involved in transcription and stress response; functions as part of a network of genes in exit from mitosis; localization is cell cycle regulated; activated by Cdc15p during the exit from mitosis [Source:SGD;Acc:S000003324] |
| comp5488_c0 | YGR144W | 3.93 | 0.05 | Thiazole synthase, catalyzes formation of a thiazole intermediate during thiamine biosynthesis; required for mitochondrial genome stability in response to DNA damaging agents [Source:SGD;Acc:S000003376] |
| comp4073_c0 | YHR073W | #NAME? | 0.03 | Member of an oxysterol-binding protein family with seven members in S. cerevisiae; family members have overlapping, redundant functions in sterol metabolism and collectively perform a function essential for viability [Source:SGD;Acc:S000001115] |
| comp499_c0 | YIL053W | 4.14 | 0.04 | Constitutively expressed isoform of DL-glycerol-3- phosphatase; involved in glycerol biosynthesis, induced in response to both anaerobic and, along with the Hor2p/Gpp2p isoform, osmotic stress [Source:SGD;Acc:S000001315] |
| comp3427_c0 | YIL124W | 3.89 | 0.05 | NADPH-dependent 1-acyl dihydroxyacetone phosphate reductase found in lipid particles, ER, and mitochondrial outer membrane; involved in phosphatidic acid biosynthesis; required for spore germination; capable of metabolizing steroid hormones [Source:SGD;Acc:S000001386] |
| comp84_c0 | YKL085W | -4.13 | 0.04 | Mitochondrial malate dehydrogenase, catalyzes interconversion of malate and oxaloacetate; involved in the tricarboxylic acid (TCA) cycle; phosphorylated [Source:SGD;Acc:S000001568] |
| comp6484_c0 | YKL090W | -5.75 | 0.03 | Protein of unknown function; has two CUE domains that bind ubiquitin, which may facilitate intramolecular monoubiquitination [Source:SGD;Acc:S000001573] |
| comp2602_c0 | YKL105C | 4.04 | 0.04 | Putative protein of unknown function [Source:SGD;Acc:S000001588] |
| comp1140_c0 | YKR039W | 5.48 | 0.01 | General amino acid permease; Gap1p senses the presence of amino acid substrates to regulate localization to the plasma membrane when needed [Source:SGD;Acc:S000001747] |
| comp2_c0 | YKR097W | -4.51 | 0.03 | Phosphoenolpyruvate carboxykinase, key enzyme in gluconeogenesis, catalyzes early reaction in carbohydrate biosynthesis, glucose represses transcription and accelerates mRNA degradation, regulated by Mcm1p and Cat8p, located in the cytosol [Source:SGD;Acc:S000001805] |
| comp189_c0 | YLR214W | -5.11 | 0.02 | Ferric reductase and cupric reductase, reduces siderophore-bound iron and oxidized copper prior to uptake by transporters; expression induced by low copper and iron levels [Source:SGD;Acc:S000004204] |
| comp1288_c0 | YML123C | 5.10 | 0.02 | High-affinity inorganic phosphate (Pi) transporter and low-affinity manganese transporter; regulated by Pho4p and Spt7p; mutation confers resistance to arsenate; exit from the ER during maturation requires Pho86p [Source:SGD;Acc:S000004592] |
| comp1223_c0 | YMR240C | -4.50 | 0.03 | Protein required for assembly of U2 snRNP into the spliceosome, forms a complex with Hsh49p and Hsh155p [Source:SGD;Acc:S000004853] |
| comp9497_c0 | YNL023C | 4.03 | 0.05 | Protein that binds to Fpr1p, conferring rapamycin resistance by competing with rapamycin for Fpr1p binding; accumulates in the nucleus upon treatment of cells with rapamycin; has similarity to D. melanogaster shuttle craft and human NFX1 [Source:SGD;Acc:S000004968] |
| comp9505_c0 | YNL141W | 4.39 | 0.04 | Adenine deaminase (adenine aminohydrolase), converts adenine to hypoxanthine; involved in purine salvage; transcriptionally regulated by nutrient levels and growth phase; Aah1p degraded upon entry into quiescence via SCF and the proteasome [Source:SGD;Acc:S000005085] |
| comp8962_c0 | YNL142W | 3.99 | 0.05 | Ammonium permease involved in regulation of pseudohyphal growth; belongs to a ubiquitous family of cytoplasmic membrane proteins that transport only ammonium (NH4+); expression is under the nitrogen catabolite repression regulation [Source:SGD;Acc:S000005086] |
| comp1364_c0 | YOR049C | 4.45 | 0.03 | Suppressor of sphingoid long chain base (LCB) sensitivity of an LCB-lyase mutation; putative integral membrane transporter or flippase that may transport LCBs from the cytoplasmic side toward the extracytoplasmic side of the membrane [Source:SGD;Acc:S000005575] |

| id | SC matching gene | log2FoldChange | pval | description |
| --- | --- | --- | --- | --- |
| Mf-fruit interaction | | | | |
| comp1736_c0 | YAL038W | 3.312073 | 0.032588 | Pyruvate kinase, functions as a homotetramer in glycolysis to convert phosphoenolpyruvate to pyruvate, the input for aerobic (TCA cycle) or anaerobic (glucose fermentation) respiration [Source:SGD;Acc:S000000036] |
| comp360_c0 | YBL045C | -3.11845 | 0.042949 | Core subunit of the ubiquinol-cytochrome c reductase complex (bc1 complex), which is a component of the mitochondrial inner membrane electron transport chain [Source:SGD;Acc:S000000141] |
| comp382_c0 | YBL064C | 3.818211 | 0.015653 | Mitochondrial peroxiredoxin (1-Cys Prx) with thioredoxin peroxidase activity, has a role in reduction of hydroperoxides; reactivation requires Trr2p and glutathione; induced during respiratory growth and oxidative stress; phosphorylated [Source:SGD;Acc:S000000160] |
| comp3650_c0 | YBL066C | 3.803344 | 0.016754 | Putative transcription factor, has homolog in Kluyveromyces lactis [Source:SGD;Acc:S000000162] |
| comp458_c0 | YBL092W | 3.244328 | 0.035524 | Protein component of the large (60S) ribosomal subunit, has similarity to rat L32 ribosomal protein; overexpression disrupts telomeric silencing [Source:SGD;Acc:S000000188] |
| comp1753_c0 | YBR094W | 4.42754 | 0.006452 | Putative tubulin tyrosine ligase associated with P- bodies [Source:SGD;Acc:S000000298] |
| comp615_c0 | YBR189W | 3.61852 | 0.020972 | Protein component of the small (40S) ribosomal subunit; nearly identical to Rps9Ap and has similarity to E. coli S4 and rat S9 ribosomal proteins [Source:SGD;Acc:S000000393] |
| comp3662_c0 | YBR294W | 3.047324 | 0.047884 | High affinity sulfate permease of the SulP anion transporter family; sulfate uptake is mediated by specific sulfate transporters Sul1p and Sul2p, which control the concentration of endogenous activated sulfate intermediates [Source:SGD;Acc:S000000498] |
| comp6609_c0 | YCL064C | 3.513262 | 0.039901 | Catabolic L-serine (L-threonine) deaminase, catalyzes the degradation of both L-serine and L- threonine; required to use serine or threonine as the sole nitrogen source, transcriptionally induced by serine and threonine [Source:SGD;Acc:S000000569] |
| comp8775_c0 | YCR098C | 4.010762 | 0.017763 | Plasma membrane permease, mediates uptake of glycerophosphoinositol and glycerophosphocholine as sources of the nutrients inositol and phosphate; expression and transport rate are regulated by phosphate and inositol availability [Source:SGD;Acc:S000000695] |
| comp473_c0 | YDL083C | 3.391654 | 0.028968 | Protein component of the small (40S) ribosomal subunit; identical to Rps16Ap and has similarity to E. coli S9 and rat S16 ribosomal proteins [Source:SGD;Acc:S000002241] |
| comp462_c0 | YDL191W | 3.578357 | 0.022248 | Protein component of the large (60S) ribosomal subunit, identical to Rpl35Bp and has similarity to rat L35 ribosomal protein [Source:SGD;Acc:S000002350] |
| comp1363_c0 | YDR044W | 3.994102 | 0.012238 | Coproporphyrinogen III oxidase, an oxygen requiring enzyme that catalyzes the sixth step in the heme biosynthetic pathway; transcription is repressed by oxygen and heme (via Rox1p and Hap1p) [Source:SGD;Acc:S000002451] |
| comp8321_c0 | YDR127W | 5.821811 | 0.001139 | Pentafunctional arom protein, catalyzes steps 2 through 6 in the biosynthesis of chorismate, which is a precursor to aromatic amino acids [Source:SGD;Acc:S000002534] |
| comp39_c0 | YDR258C | -3.18198 | 0.038451 | Oligomeric mitochondrial matrix chaperone that cooperates with Ssc1p in mitochondrial thermotolerance after heat shock; able to prevent the aggregation of misfolded proteins as well as resolubilize protein aggregates [Source:SGD;Acc:S000002666] |
| comp6779_c0 | YDR345C | 4.600917 | 0.005573 | Low affinity glucose transporter of the major facilitator superfamily, expression is induced in low or high glucose conditions [Source:SGD;Acc:S000002753] |
| comp443_c0 | YDR497C | -3.07586 | 0.045448 | Myo-inositol transporter with strong similarity to the minor myo-inositol transporter Itr2p, member of the sugar transporter superfamily; expression is repressed by inositol and choline via Opi1p and derepressed via Ino2p and Ino4p [Source:SGD;Acc:S000002905] |
| comp538_c0 | YDR500C | 3.06116 | 0.046046 | Protein component of the large (60S) ribosomal subunit, has similarity to Rpl37Ap and to rat L37 ribosomal protein [Source:SGD;Acc:S000002908] |
| comp334_c0 | YDR536W | -3.55598 | 0.023371 | Glycerol proton symporter of the plasma membrane, subject to glucose-induced inactivation, strongly but transiently induced when cells are subjected to osmotic shock [Source:SGD;Acc:S000002944] |
| comp8511_c0 | YEL052W | 3.264991 | 0.048697 | Conserved protein that may act as a chaperone in the degradation of misfolded or unassembled cytochrome c oxidase subunits; localized to matrix face of the mitochondrial inner membrane; member of the AAA family but lacks a protease domain [Source:SGD;Acc:S000000778] |
| comp401_c0 | YEL054C | 2.998758 | 0.049593 | Protein component of the large (60S) ribosomal subunit, nearly identical to Rpl12Bp; rpl12a rpl12b double mutant exhibits slow growth and slow translation; has similarity to E. coli L11 and rat L12 ribosomal proteins [Source:SGD;Acc:S000000780] |
| comp7737_c0 | YEL065W | 4.310358 | 0.009995 | Ferrioxamine B transporter, member of the ARN family of transporters that specifically recognize siderophore-iron chelates; transcription is induced during iron deprivation and diauxic shift; potentially phosphorylated by Cdc28p [Source:SGD;Acc:S000000791] |
| comp3927_c0 | YEL065W | 3.133906 | 0.049626 | Ferrioxamine B transporter, member of the ARN family of transporters that specifically recognize siderophore-iron chelates; transcription is induced during iron deprivation and diauxic shift; potentially phosphorylated by Cdc28p [Source:SGD;Acc:S000000791] |
| comp4109_c0 | YER145C | 5.706088 | 0.001038 | High affinity iron permease involved in the transport of iron across the plasma membrane; forms complex with Fet3p; expression is regulated by iron [Source:SGD;Acc:S000000947] |
| comp4185_c0 | YER145C | 5.46214 | 0.001466 | High affinity iron permease involved in the transport of iron across the plasma membrane; forms complex with Fet3p; expression is regulated by iron [Source:SGD;Acc:S000000947] |
| comp4227_c0 | YER145C | 4.346386 | 0.009366 | High affinity iron permease involved in the transport of iron across the plasma membrane; forms complex with Fet3p; expression is regulated by iron [Source:SGD;Acc:S000000947] |
| comp6052_c0 | YFR030W | 3.792507 | 0.018432 | Subunit alpha of assimilatory sulfite reductase, which converts sulfite into sulfide [Source:SGD;Acc:S000001926] |
| comp721_c0 | YFR032C-A | 3.370279 | 0.030385 | Protein component of the large (60S) ribosomal subunit, has similarity to rat L29 ribosomal protein; not essential for translation, but required for proper joining of the large and small ribosomal subunits and for normal translation rate [Source:SGD;Acc:S000006437] |
| comp537_c0 | YGL030W | 3.347023 | 0.030998 | Protein component of the large (60S) ribosomal subunit, has similarity to rat L30 ribosomal protein; involved in pre-rRNA processing in the nucleolus; autoregulates splicing of its transcript [Source:SGD;Acc:S000002998] |
| comp590_c0 | YGL076C | 3.351055 | 0.030532 | Protein component of the large (60S) ribosomal subunit, nearly identical to Rpl7Bp and has similarity to E. coli L30 and rat L7 ribosomal proteins; contains a conserved C-terminal Nucleic acid Binding Domain (NDB2) [Source:SGD;Acc:S000003044] |
| comp2116_c0 | YGR092W | -3.40112 | 0.034759 | Ser/Thr kinase involved in transcription and stress response; functions as part of a network of genes in exit from mitosis; localization is cell cycle regulated; activated by Cdc15p during the exit from mitosis [Source:SGD;Acc:S000003324] |
| comp5488_c0 | YGR144W | 3.244832 | 0.041898 | Thiazole synthase, catalyzes formation of a thiazole intermediate during thiamine biosynthesis; required for mitochondrial genome stability in response to DNA damaging agents [Source:SGD;Acc:S000003376] |
| comp2062_c0 | YGR175C | 3.126199 | 0.042457 | Squalene epoxidase, catalyzes the epoxidation of squalene to 2,3-oxidosqualene; plays an essential role in the ergosterol-biosynthesis pathway and is the specific target of the antifungal drug terbinafine [Source:SGD;Acc:S000003407] |
| comp680_c0 | YHR007C | 3.356648 | 0.030229 | Lanosterol 14-alpha-demethylase, catalyzes the C-14 demethylation of lanosterol to form 4,4''-dimethyl cholesta-8,14,24-triene-3-beta-ol in the ergosterol biosynthesis pathway; member of the cytochrome P450 family [Source:SGD;Acc:S000001049] |
| comp3730_c0 | YHR094C | 3.477089 | 0.036119 | Low-affinity glucose transporter of the major facilitator superfamily, expression is induced by Hxk2p in the presence of glucose and repressed by Rgt1p when glucose is limiting [Source:SGD;Acc:S000001136] |
| comp420_c0 | YIL018W | 3.015945 | 0.048427 | Protein component of the large (60S) ribosomal subunit, identical to Rpl2Ap and has similarity to E. coli L2 and rat L8 ribosomal proteins; expression is upregulated at low temperatures [Source:SGD;Acc:S000001280] |
| comp552_c0 | YIL052C | 3.195938 | 0.038107 | Protein component of the large (60S) ribosomal subunit, nearly identical to Rpl34Ap and has similarity to rat L34 ribosomal protein [Source:SGD;Acc:S000001314] |
| comp8989_c0 | YIL160C | 5.804866 | 0.001312 | 3-ketoacyl-CoA thiolase with broad chain length specificity, cleaves 3-ketoacyl-CoA into acyl-CoA and acetyl-CoA during beta-oxidation of fatty acids [Source:SGD;Acc:S000001422] |
| comp10168_c0 | YIL160C | 4.663822 | 0.009407 | 3-ketoacyl-CoA thiolase with broad chain length specificity, cleaves 3-ketoacyl-CoA into acyl-CoA and acetyl-CoA during beta-oxidation of fatty acids [Source:SGD;Acc:S000001422] |
| comp3361_c0 | YIR019C | 6.39936 | 0.000314 | GPI-anchored cell surface glycoprotein (flocculin) required for pseudohyphal formation, invasive growth, flocculation, and biofilms; transcriptionally regulated by the MAPK pathway (via Ste12p and Tec1p) and the cAMP pathway (via Flo8p) [Source:SGD;Acc:S000001458] |
| comp2822_c0 | YJL012C | 3.051931 | 0.047864 | Vacuolar membrane polyphosphate polymerase; subunit of the vacuolar transporter chaperone (VTC) complex involved in synthesis and transfer of polyP to the vacuole; regulates membrane trafficking; role in non- autophagic vacuolar fusion [Source:SGD;Acc:S000003549] |
| comp4688_c0 | YJL026W | 5.209256 | 0.002302 | Ribonucleotide-diphosphate reductase (RNR), small subunit; the RNR complex catalyzes the rate-limiting step in dNTP synthesis and is regulated by DNA replication and DNA damage checkpoint pathways via localization of the small subunits [Source:SGD;Acc:S000003563] |
| comp8747_c0 | YJL026W | 4.718029 | 0.006123 | Ribonucleotide-diphosphate reductase (RNR), small subunit; the RNR complex catalyzes the rate-limiting step in dNTP synthesis and is regulated by DNA replication and DNA damage checkpoint pathways via localization of the small subunits [Source:SGD;Acc:S000003563] |
| comp6081_c0 | YJL026W | 4.451201 | 0.008732 | Ribonucleotide-diphosphate reductase (RNR), small subunit; the RNR complex catalyzes the rate-limiting step in dNTP synthesis and is regulated by DNA replication and DNA damage checkpoint pathways via localization of the small subunits [Source:SGD;Acc:S000003563] |
| comp632_c0 | YJL189W | 3.517156 | 0.024674 | Protein component of the large (60S) ribosomal subunit, has similarity to rat L39 ribosomal protein; required for ribosome biogenesis; loss of both Rpl31p and Rpl39p confers lethality; also exhibits genetic interactions with SIS1 and PAB1 [Source:SGD;Acc:S000003725] |
| comp93_c0 | YJR045C | -3.16792 | 0.039714 | Hsp70 family ATPase, constituent of the import motor component of the Translocase of the Inner Mitochondrial membrane (TIM23 complex); involved in protein translocation and folding; subunit of SceI endonuclease [Source:SGD;Acc:S000003806] |
| comp4922_c0 | YJR070C | 3.153751 | 0.043097 | Deoxyhypusine hydroxylase, a HEAT-repeat containing metalloenzyme that catalyzes hypusine formation; binds to and is required for the modification of Hyp2p (eIF5A); complements S. pombe mmd1 mutants defective in mitochondrial positioning [Source:SGD;Acc:S000003831] |
| comp2126_c0 | YJR078W | 3.310703 | 0.032774 | Putative tryptophan 2,3-dioxygenase or indoleamine 2,3-dioxygenase, required for de novo biosynthesis of NAD from tryptophan via kynurenine; interacts genetically with telomere capping gene CDC13; regulated by Hst1p and Aftp [Source:SGD;Acc:S000003839] |
| comp4738_c0 | YJR109C | 3.245782 | 0.042914 | Large subunit of carbamoyl phosphate synthetase, which catalyzes a step in the synthesis of citrulline, an arginine precursor [Source:SGD;Acc:S000003870] |
| comp620_c0 | YJR123W | 3.134296 | 0.041321 | Protein component of the small (40S) ribosomal subunit, the least basic of the non-acidic ribosomal proteins; phosphorylated in vivo; essential for viability; has similarity to E. coli S7 and rat S5 ribosomal proteins [Source:SGD;Acc:S000003884] |
| comp2225_c0 | YJR137C | 3.17374 | 0.039618 | Sulfite reductase beta subunit, involved in amino acid biosynthesis, transcription repressed by methionine [Source:SGD;Acc:S000003898] |
| comp2092_c0 | YKL009W | 3.013796 | 0.049789 | Protein involved in mRNA turnover and ribosome assembly, localizes to the nucleolus [Source:SGD;Acc:S000001492] |
| comp84_c0 | YKL085W | -4.01894 | 0.011801 | Mitochondrial malate dehydrogenase, catalyzes interconversion of malate and oxaloacetate; involved in the tricarboxylic acid (TCA) cycle; phosphorylated [Source:SGD;Acc:S000001568] |
| comp104_c0 | YKL125W | -3.72299 | 0.018011 | Protein required for transcription of rDNA by RNA polymerase I; transcription factor independent of DNA template; involved in recruitment of RNA polymerase I to rDNA [Source:SGD;Acc:S000001608] |
| comp2107_c0 | YKL175W | 3.941961 | 0.013519 | Vacuolar membrane zinc transporter, transports zinc from storage in the vacuole to the cytoplasm when needed; transcription is induced under conditions of zinc deficiency [Source:SGD;Acc:S000001658] |
| comp4643_c0 | YKL215C | -4.42827 | 0.034118 | 5-oxoprolinase; enzyme is ATP-dependent and functions as a dimer; similar to mouse Oplah gene; green fluorescent protein (GFP)-fusion protein localizes to the cytoplasm [Source:SGD;Acc:S000001698] |
| comp6363_c0 | YKL215C | -4.48815 | 0.050584 | 5-oxoprolinase; enzyme is ATP-dependent and functions as a dimer; similar to mouse Oplah gene; green fluorescent protein (GFP)-fusion protein localizes to the cytoplasm [Source:SGD;Acc:S000001698] |
| comp1140_c0 | YKR039W | 4.240293 | 0.008466 | General amino acid permease; Gap1p senses the presence of amino acid substrates to regulate localization to the plasma membrane when needed [Source:SGD;Acc:S000001747] |
| comp2756_c0 | YKR043C | 3.303859 | 0.034303 | Metal-independent fructose 1,6-bisphosphatase, may play a role in gluconeogenesis under metal-limiting conditions; green fluorescent protein (GFP)-fusion protein localizes to the cytoplasm and nucleus [Source:SGD;Acc:S000001751] |
| comp13_c0 | YLL026W | -3.17129 | 0.038962 | Heat shock protein that cooperates with Ydj1p (Hsp40) and Ssa1p (Hsp70) to refold and reactivate previously denatured, aggregated proteins; responsive to stresses including: heat, ethanol, and sodium arsenite; involved in [PSI+] propagation [Source:SGD;Acc:S000003949] |
| comp2015_c0 | YLL028W | -3.84109 | 0.018088 | Polyamine transporter that recognizes spermine, putrescine, and spermidine; catalyzes uptake of polyamines at alkaline pH and excretion at acidic pH; phosphorylation enhances activity and sorting to the plasma membrane [Source:SGD;Acc:S000003951] |
| comp220_c0 | YLL039C | 3.553668 | 0.023022 | Ubiquitin, becomes conjugated to proteins, marking them for selective degradation via the ubiquitin-26S proteasome system; essential for the cellular stress response; encoded as a polyubiquitin precursor comprised of 5 head-to-tail repeats [Source:SGD;Acc:S000003962] |
| comp8411_c0 | YLL057C | 6.881006 | 0.000168 | Fe(II)-dependent sulfonate/alpha-ketoglutarate dioxygenase, involved in sulfonate catabolism for use as a sulfur source; contains sequence that resembles a J domain (typified by the E. coli DnaJ protein); induced by sulphur starvation [Source:SGD;Acc:S000003980] |
| comp9471_c0 | YLL057C | 6.353222 | 0.000469 | Fe(II)-dependent sulfonate/alpha-ketoglutarate dioxygenase, involved in sulfonate catabolism for use as a sulfur source; contains sequence that resembles a J domain (typified by the E. coli DnaJ protein); induced by sulphur starvation [Source:SGD;Acc:S000003980] |
| comp6312_c0 | YLL057C | 6.09596 | 0.000668 | Fe(II)-dependent sulfonate/alpha-ketoglutarate dioxygenase, involved in sulfonate catabolism for use as a sulfur source; contains sequence that resembles a J domain (typified by the E. coli DnaJ protein); induced by sulphur starvation [Source:SGD;Acc:S000003980] |
| comp236_c0 | YLR044C | 4.124742 | 0.009972 | Major of three pyruvate decarboxylase isozymes, key enzyme in alcoholic fermentation, decarboxylates pyruvate to acetaldehyde; subject to glucose-, ethanol-, and autoregulation; involved in amino acid catabolism [Source:SGD;Acc:S000004034] |
| comp2088_c0 | YLR056W | 5.364721 | 0.001549 | C-5 sterol desaturase, catalyzes the introduction of a C-5(6) double bond into episterol, a precursor in ergosterol biosynthesis; mutants are viable, but cannot grow on non-fermentable carbon sources [Source:SGD;Acc:S000004046] |
| comp675_c0 | YLR061W | 3.394461 | 0.029014 | Protein component of the large (60S) ribosomal subunit, has similarity to Rpl22Bp and to rat L22 ribosomal protein [Source:SGD;Acc:S000004051] |
| comp164_c0 | YLR121C | 3.582868 | 0.021896 | Aspartic protease, member of the yapsin family of proteases involved in cell wall growth and maintenance; attached to the plasma membrane via a glycosylphosphatidylinositol (GPI) anchor [Source:SGD;Acc:S000004111] |
| comp3709_c0 | YLR130C | 5.909253 | 0.000684 | Low-affinity zinc transporter of the plasma membrane; transcription is induced under low-zinc conditions by the Zap1p transcription factor [Source:SGD;Acc:S000004120] |
| comp1369_c0 | YLR136C | 3.421106 | 0.027982 | mRNA-binding protein expressed during iron starvation; binds to a sequence element in the 3'- untranslated regions of specific mRNAs to mediate their degradation; involved in iron homeostasis [Source:SGD;Acc:S000004126] |
| comp189_c0 | YLR214W | -5.35958 | 0.001623 | Ferric reductase and cupric reductase, reduces siderophore-bound iron and oxidized copper prior to uptake by transporters; expression induced by low copper and iron levels [Source:SGD;Acc:S000004204] |
| comp344_c0 | YLR216C | -3.05659 | 0.046691 | Peptidyl-prolyl cis-trans isomerase (cyclophilin), catalyzes the cis-trans isomerization of peptide bonds N- terminal to proline residues; binds to Hsp82p and contributes to chaperone activity [Source:SGD;Acc:S000004206] |
| comp64_c0 | YLR259C | -3.47949 | 0.025444 | Tetradecameric mitochondrial chaperonin required for ATP-dependent folding of precursor polypeptides and complex assembly; prevents aggregation and mediates protein refolding after heat shock; role in mtDNA transmission; phosphorylated [Source:SGD;Acc:S000004249] |
| comp399_c0 | YLR333C | 3.254287 | 0.035084 | Protein component of the small (40S) ribosomal subunit; nearly identical to Rps25Ap and has similarity to rat S25 ribosomal protein [Source:SGD;Acc:S000004325] |
| comp2472_c0 | YLR367W | 3.582953 | 0.024966 | Protein component of the small (40S) ribosomal subunit; nearly identical to Rps22Ap and has similarity to E. coli S8 and rat S15a ribosomal proteins [Source:SGD;Acc:S000004359] |
| comp720_c0 | YLR388W | 3.28426 | 0.034434 | Protein component of the small (40S) ribosomal subunit; nearly identical to Rps29Bp and has similarity to rat S29 and E. coli S14 ribosomal proteins [Source:SGD;Acc:S000004380] |
| comp368_c0 | YML063W | 3.332527 | 0.031254 | Ribosomal protein 10 (rp10) of the small (40S) subunit; nearly identical to Rps1Ap and has similarity to rat S3a ribosomal protein [Source:SGD;Acc:S000004528] |
| comp1108_c0 | YMR063W | 2.990502 | 0.050476 | Protein of unknown function, involved in the proteolytic activation of Rim101p in response to alkaline pH; has similarity to A. nidulans PalI; putative membrane protein [Source:SGD;Acc:S000004667] |
| comp1311_c0 | YMR086W | -3.19848 | 0.041031 | Component of the eisosome with unknown function; may interact with ribosomes, based on co-purification experiments; GFP-fusion protein localizes to the cell periphery; expression is repressed by cAMP [Source:SGD;Acc:S000004692] |
| comp292_c0 | YMR121C | 3.095359 | 0.043366 | Protein component of the large (60S) ribosomal subunit, nearly identical to Rpl15Ap and has similarity to rat L15 ribosomal protein; binds to 5.8 S rRNA [Source:SGD;Acc:S000004728] |
| comp4008_c0 | YMR169C | -3.75508 | 0.048401 | Cytoplasmic aldehyde dehydrogenase, involved in beta-alanine synthesis; uses NAD+ as the preferred coenzyme; very similar to Ald2p; expression is induced by stress and repressed by glucose [Source:SGD;Acc:S000004779] |
| comp477_c0 | YMR189W | -4.37358 | 0.007232 | P subunit of the mitochondrial glycine decarboxylase complex, required for the catabolism of glycine to 5,10-methylene-THF; expression is regulated by levels of 5,10-methylene-THF in the cytoplasm [Source:SGD;Acc:S000004801] |
| comp1223_c0 | YMR240C | -5.65416 | 0.001569 | Protein required for assembly of U2 snRNP into the spliceosome, forms a complex with Hsh49p and Hsh155p [Source:SGD;Acc:S000004853] |
| comp87_c0 | YNL007C | -3.67823 | 0.019313 | Type II HSP40 co-chaperone that interacts with the HSP70 protein Ssa1p; not functionally redundant with Ydj1p due to due to substrate specificity; shares similarity with bacterial DnaJ proteins [Source:SGD;Acc:S000004952] |
| comp325_c0 | YNL069C | 3.07435 | 0.044693 | N-terminally acetylated protein component of the large (60S) ribosomal subunit, binds to 5.8 S rRNA; has similarity to Rpl16Ap, E. coli L13 and rat L13a ribosomal proteins; transcriptionally regulated by Rap1p [Source:SGD;Acc:S000005013] |
| comp9505_c0 | YNL141W | 3.823602 | 0.034253 | Adenine deaminase (adenine aminohydrolase), converts adenine to hypoxanthine; involved in purine salvage; transcriptionally regulated by nutrient levels and growth phase; Aah1p degraded upon entry into quiescence via SCF and the proteasome [Source:SGD;Acc:S000005085] |
| comp705_c0 | YNL162W | 3.082202 | 0.044715 | Protein component of the large (60S) ribosomal subunit, identical to Rpl42Bp and has similarity to rat L44 ribosomal protein [Source:SGD;Acc:S000005106] |
| comp396_c0 | YNL209W | 3.071102 | 0.044832 | Cytoplasmic ATPase that is a ribosome-associated molecular chaperone, functions with J-protein partner Zuo1p; may be involved in the folding of newly-synthesized polypeptide chains; member of the HSP70 family; homolog of SSB1 [Source:SGD;Acc:S000005153] |
| comp10376_c0 | YNL217W | 3.569846 | 0.034015 | Putative protein of unknown function; weak sequence similarity to bis (5'-nucleotidyl)-tetraphosphatases; (GFP)-fusion protein localizes to the vacuole; null mutant is highly sensitive to azaserine and resistant to sodium- O-vandate [Source:SGD;Acc:S000005161] |
| comp5339_c0 | YNL239W | 4.698072 | 0.007126 | Cysteine aminopeptidase with homocysteine- thiolactonase activity; protects cells against homocysteine toxicity; has bleomycin hydrolase activity in vitro; transcription is regulated by galactose via Gal4p; orthologous to human BLMH [Source:SGD;Acc:S000005183] |
| comp2748_c0 | YNR046W | 3.186293 | 0.041899 | Subunit of tRNA methyltransferase (MTase) complexes in combination with Trm9p and Trm11p; subunit of complex with Mtq2p that methylates Sup45p (eRF1) in the ternary complex eRF1-eRF3-GTP; deletion confers resistance to zymocin [Source:SGD;Acc:S000005329] |
| comp3295_c0 | YNR062C | 5.303917 | 0.001749 | Putative membrane protein of unknown function [Source:SGD;Acc:S000005345] |
| comp7584_c0 | YNR062C | 5.459663 | 0.001853 | Putative membrane protein of unknown function [Source:SGD;Acc:S000005345] |
| comp5062_c0 | YOL103W | 3.848446 | 0.016788 | Myo-inositol transporter with strong similarity to the major myo-inositol transporter Itr1p, member of the sugar transporter superfamily; expressed constitutively [Source:SGD;Acc:S000005463] |
| comp283_c0 | YOL155C | -3.93873 | 0.013358 | Haze-protective mannoprotein that reduces the particle size of aggregated proteins in white wines [Source:SGD;Acc:S000005515] |
| comp410_c0 | YOR007C | -3.2522 | 0.035824 | Glutamine-rich cytoplasmic protein that serves as a scaffold for binding Get4/5p and other proteins required to mediate posttranslational insertion of tail-anchored proteins into the ER membrane; has similarity to human cochaperone SGT [Source:SGD;Acc:S000005533] |
| comp7996_c0 | YOR252W | 3.266622 | 0.044805 | Protein of unknown function that associates with ribosomes [Source:SGD;Acc:S000005778] |
| comp2220_c0 | YOR294W | 3.048688 | 0.047779 | Essential protein that binds ribosomal protein L11 and is required for nuclear export of the 60S pre- ribosomal subunit during ribosome biogenesis; mouse homolog shows altered expression in Huntington's disease model mice [Source:SGD;Acc:S000005820] |
| comp544_c0 | YOR312C | 3.577488 | 0.022234 | Protein component of the large (60S) ribosomal subunit, nearly identical to Rpl20Ap and has similarity to rat L18a ribosomal protein [Source:SGD;Acc:S000005839] |
| comp979_c0 | YOR369C | 3.261423 | 0.034996 | Protein component of the small (40S) ribosomal subunit; has similarity to rat ribosomal protein S12 [Source:SGD;Acc:S000005896] |
| comp776_c0 | YPL111W | -4.12222 | 0.011546 | Arginase, responsible for arginine degradation, expression responds to both induction by arginine and nitrogen catabolite repression; disruption enhances freeze tolerance [Source:SGD;Acc:S000006032] |
| comp1700_c0 | YPL170W | 3.034581 | 0.049267 | Heme-binding protein involved in regulation of cytochrome P450 protein Erg11p; damage response protein, related to mammalian membrane progesterone receptors; mutations lead to defects in telomeres, mitochondria, and sterol synthesis [Source:SGD;Acc:S000006091] |
| comp661_c1 | YPL179W | 3.194982 | 0.038477 | Putative protein serine/threonine phosphatase; null mutation enhances efficiency of translational suppressors [Source:SGD;Acc:S000006100] |
| comp5533_c0 | YPR194C | 3.493438 | 0.027269 | Oligopeptide transporter; member of the OPT family, with potential orthologs in S. pombe and C. albicans; also plays a role in formation of mature vacuoles [Source:SGD;Acc:S000006398] |
