# Supplementary material for: De-novo assembly and characterization of the transcriptome of Metschnikowia fructicola reveals differences in gene expression following interaction with Penicillium digitatum and grapefruit peel
Source: BMC Genomics. 2013 Mar 12;14:168. doi: 10.1186/1471-2164-14-168 (PMC3608080; doi:10.1186/1471-2164-14-168)
Supplement: Additional file 5 — Summary of differential expressed genes in Metschnikowia fructicola interaction with Penicillium digitatum and interaction with fruit (p < 0.05) involved in the response to stresses (chemical GO:042221), oxidative (GO:006979), osmotic (GO:006970), heat (GO:009408), starvation (GO:042594), DNA damage stimulus (GO:006974). [file 1471-2164-14-168-S5.docx]

***De-novo* assembly and characterization of the transcriptome of *Metschnikowia fructicola* reveals differences in gene expression following interaction with *Penicillium digitatum* and grapefruit peel**

**Vera Hershkovitz^1,^** **^†^**

Email: vhershko@agri.gov.il

**Noa Sela^2, †^**

Email: [noa@agri.gov.il](mailto:noa@agri.gov.il)

**Leena Taha-Salaime^1,3,4^**

Email: [leena.taha@mail.huji.ac.il](mailto:leena.taha@mail.huji.ac.il)

**Jia Liu^5^**

Email:Jia.Liu@ARS.USDA.GOV

**Ginat Rafael^1^**

Email: [pongie@volcani.agri.gov.il](mailto:pongie@volcani.agri.gov.il)

**Clarita Kessler^1^**

Email: [clarita.bendayan@gmail.com](mailto:clarita.bendayan@gmail.com)

**Radi Aly^3^**

Email: [radi@volcani.agri.gov.il](mailto:radi@volcani.agri.gov.il)

**Maggie Levy^4^**

Email: [levym@agri.huji.ac.il](mailto:levym@agri.huji.ac.il)

**Michael Wisniewski^5^**

Email: Michael.Wisniewski@ARS.USDA.GOV

**Samir Droby^1*^**

* Corresponding author

Email: samird[@volcani.agri.gov.il](mailto:samird@volcani.agri.gov.il)

**^1^** Department of Postharvest and Food Sciences, ARO, the Volcani Center, Bet Dagan 50250, Israel

^2^ Department of Plant Pathology and Weed Research, ARO, the Volcani Center, Bet Dagan 50250, Israel

^3^ Department of Plant Pathology and Weed Research, the Volcani Center, Newe-Yaar Research Center, Israel.

^4^ [Department of Plant Pathology and Microbiology](http://departments.agri.huji.ac.il/plantpath/), [the Robert H. Smith Faculty of Agriculture, Food and Environment ,](http://www.agri.huji.ac.il/) [the Hebrew University of Jerusalem](http://www.huji.ac.il/), Israel.

^5^ U.S. Department of Agriculture-Agricultural Research Service (USDA-ARS), Appalachian Fruit Research Station, WV, USA.

† Equal contributors.

**Table 3**. Summary of differential expressed genes in M. fructicola interaction with P. digitatum and interaction with fruit ( *p* < 0.05) involved in the response to stresses (chemical GO:042221), oxidative (GO:006979, 0055114 ), osmotic (GO:006970), heat (GO:009408), starvation (GO:042594), DNA damage stimulus (GO:006974).

|  |  |  |  |  |  |
| --- | --- | --- | --- | --- | --- |
| Standard name | Systematic name | contig | Description | Log2 FC Pdig | log FC fruit |
| **response to chemical stimulus (GO:042221)** | | | | | |
| SNF3 | YDL194W | comp928_c0 | Plasma membrane low glucose sensor that regulates glucose transport; contains 12 predicted transmembrane segments and a long C-terminal tail required for induction of hexose transporters; also senses fructose and mannose; similar to Rgt2p | 4.2 | - |
| YHK8 | YHR048W | comp6236_c0 | Presumed antiporter of the DHA1 family of multidrug resistance transporters; contains 12 predicted transmembrane spans; expression of gene is up-regulated in cells exhibiting reduced susceptibility to azoles | 3.4 | - |
| YOR1 | YGR281W | comp2447_c0 | Plasma membrane ATP-binding cassette (ABC) transporter, multidrug transporter mediates export of many different organic anions including oligomycin; similar to human cystic fibrosis transmembrane receptor (CFTR) | 2.5 | - |
| SNQ2 | YDR011W | comp6782_c0 | Plasma membrane ATP-binding cassette (ABC) transporter, multidrug transporter involved in multidrug resistance and resistance to singlet oxygen species | 2.7 | - |
| HSP104 | YLL026W | comp13_c0 | Heat shock protein that cooperates with Ydj1p (Hsp40) and Ssa1p (Hsp70) to refold and reactivate previously denatured, aggregated proteins; responsive to stresses including: heat, ethanol, and sodium arsenite; involved in [PSI+] propagation | -1.8 | -3.2 |
| FET3 | YMR058W | comp1589_c0 | Ferro-O2-oxidoreductase required for high-affinity iron uptake and involved in mediating resistance to copper ion toxicity, belongs to class of integral membrane multicopper oxidases | - | 2.7 |
| PRX1 | YBL064C | comp382_c0 | Mitochondrial peroxiredoxin (1-Cys Prx) with thioredoxin peroxidase activity, has a role in reduction of hydroperoxides; reactivation requires Trr2p and glutathione; induced during respiratory growth and oxidative stress; phosphorylated | - | 3.8 |
| LAP3 | YNL239W | comp5339_c0 | Cysteine aminopeptidase with homocysteine- thiolactonase activity; protects cells against homocysteine toxicity; has bleomycin hydrolase activity in vitro; transcription is regulated by galactose via Gal4p | - | 4.7 |
| **response to oxidative stress (006979) and oxidation-reduction (0055114)** | | | | | |
|  | NA | comp711_c0 |  |  |  |
| MET10 | YFR030W | comp6052_c0 | Subunit alpha of assimilatory sulfite reductase, which converts sulfite into sulfide |  | 3.9 |
| HEM13 | YDR044W | comp1363_c0 | Coproporphyrinogen III oxidase, an oxygen requiring enzyme that catalyzes the sixth step in the heme biosynthetic pathway; transcription is repressed by oxygen and heme (via Rox1p and Hap1p) |  | 3.8 |
| JLP1 | YLL057C | comp8411_c0 | Fe(II)-dependent sulfonate/alpha-ketoglutarate dioxygenase, involved in sulfonate catabolism for use as a sulfur source |  | 6.3 |
| MET5 | YJR137C | comp2225_c0 | Sulfite reductase beta subunit, involved in amino acid biosynthesis, transcription repressed by methionine |  | 6.8 |
| RNR2 | YJL026W | comp4688_c0 | Ribonucleotide-diphosphate reductase (RNR), small subunit; the RNR complex catalyzes the rate-limiting step in dNTP synthesis and is regulated by DNA replication and DNA damage checkpoint pathways via localization of the small subunits |  | 4.4 |
|  | NA | comp1412_c0 |  |  | 5.2 |
| PRX1 | YBL064C | comp382_c0 | Mitochondrial peroxiredoxin (1-Cys Prx) with thioredoxin peroxidase activity, has a role in reduction of hydroperoxides; reactivation requires Trr2p and glutathione; induced during respiratory growth and oxidative stress |  | 3.5 |
| AFG1 | YEL052W | comp8511_c0 | Conserved protein that may act as a chaperone in the degradation of misfolded or unassembled cytochrome c oxidase subunits; localized to matrix face of the mitochondrial inner membrane; member of the AAA family but lacks a protease domain | - | 3.3 |
| STB5 | YHR178W | comp7125_c0 | Transcription factor, involved in regulating multidrug resistance and oxidative stress response; forms a heterodimer with Pdr1p; contains a Zn(II)2Cys6 zinc finger domain that interacts with a pleiotropic drug resistance element in vitro | 1.8 | - |
| SOD1 | YJR104C | comp513_c0 | Cytosolic copper-zinc superoxide dismutase | -2.9 | - |
| HSP104 | YLL026W | comp13_c0 | Heat shock protein that cooperates with Ydj1p (Hsp40) and Ssa1p (Hsp70) to refold and reactivate previously denatured, aggregated proteins; responsive to stresses including: heat, ethanol, and sodium arsenite | -1.8 | -3.2 |
|  |  |  |  |  |  |
|  |  |  | **response to osmotic stress (006970)** |  |  |
| RHR2 | YIL053W | comp499_c0 | Constitutively expressed isoform of DL-glycerol-3- phosphatase; involved in glycerol biosynthesis, induced in response to both anaerobic and, along with the Hor2p/Gpp2p isoform, osmotic stress | 2.4 |  |
| ISC1 | YER019W | comp2610_c0 | Mitochondrial membrane localized inositol phosphosphingolipid phospholipase C, hydrolyzes complex sphingolipids to produce ceramide; activated by phosphatidylserine, cardiolipin, and phosphatidylglycerol; mediates Na+ and Li+ halotolerance | -2.5 |  |
|  |  |  | **response to heat stress (GO: 009408)** |  |  |
| HSP78 | YDR258C | comp39_c0 | Oligomeric mitochondrial matrix chaperone that cooperates with Ssc1p in mitochondrial thermotolerance after heat shock; able to prevent the aggregation of misfolded proteins as well as resolubilize protein aggregates | -2.4 | -3.2 |
| HSP104 | YLL026W | comp13_c0 | Heat shock protein that cooperates with Ydj1p (Hsp40) and Ssa1p (Hsp70) to refold and reactivate previously denatured, aggregated proteins; responsive to stresses including: heat, ethanol, and sodium arsenite | -1.8 | -3.2 |
| SGT2 | YOR007C | comp410_c0 | Glutamine-rich cytoplasmic protein that serves as a scaffold for binding Get4/5p and other proteins required to mediate posttranslational insertion of tail-anchored proteins into the ER membrane | - | -3.3 |
| STE11 | YLR362W | comp4269_c0 | Signal transducing MEK kinase involved in pheromone response and pseudohyphal/invasive growth pathways where it phosphorylates Ste7p, and the high osmolarity response pathway, via phosphorylation of Pbs2p | -1.9 | - |
|  |  |  | **response to starvation (GO: 0042594)** |  |  |
| PHO5 | YBR093C | comp10394_c0 | Repressible acid phosphatase (1 of 3) that also mediates extracellular nucleotide-derived phosphate hydrolysis; secretory pathway derived cell surface glycoprotein; induced by phosphate starvation and coordinately regulated by PHO4 and PHO2 | 3.2 |  |
| SSB2 | YNL209W | comp396_c0 | Cytoplasmic ATPase that is a ribosome-associated molecular chaperone, functions with J-protein partner Zuo1p; may be involved in the folding of newly-synthesized polypeptide chains; member of the HSP70 family; homolog of SSB1 |  | 3.1 |
