# Supplementary material for: De-novo assembly and characterization of the transcriptome of Metschnikowia fructicola reveals differences in gene expression following interaction with Penicillium digitatum and grapefruit peel
Source: BMC Genomics. 2013 Mar 12;14:168. doi: 10.1186/1471-2164-14-168 (PMC3608080; doi:10.1186/1471-2164-14-168)
Supplement: Additional file 6 — Summary of differential expressed genes in Metschnikowia fructicola interaction with Penicillium digitatum and interaction with fruit (p < 0.05) involved in transport (transmembrane GO:055085), ion (GO:006811), carbohydrate (GO:008643), lipid (GO:006869), amino acid transport (GO:006865). [file 1471-2164-14-168-S6.docx]

***De-novo* assembly and characterization of the transcriptome of *Metschnikowia fructicola* reveals differences in gene expression following interaction with *Penicillium digitatum* and grapefruit peel**

**Vera Hershkovitz^1,^** **^†^**

Email: vhershko@agri.gov.il

**Noa Sela^2, †^**

Email: [noa@agri.gov.il](mailto:noa@agri.gov.il)

**Leena Taha-Salaime^1,3,4^**

Email: [leena.taha@mail.huji.ac.il](mailto:leena.taha@mail.huji.ac.il)

**Jia Liu^5^**

Email:Jia.Liu@ARS.USDA.GOV

**Ginat Rafael^1^**

Email: [pongie@volcani.agri.gov.il](mailto:pongie@volcani.agri.gov.il)

**Clarita Kessler^1^**

Email: [clarita.bendayan@gmail.com](mailto:clarita.bendayan@gmail.com)

**Radi Aly^3^**

Email: [radi@volcani.agri.gov.il](mailto:radi@volcani.agri.gov.il)

**Maggie Levy^4^**

Email: [levym@agri.huji.ac.il](mailto:levym@agri.huji.ac.il)

**Michael Wisniewski^5^**

Email: Michael.Wisniewski@ARS.USDA.GOV

**Samir Droby^1*^**

* Corresponding author

Email: samird[@volcani.agri.gov.il](mailto:samird@volcani.agri.gov.il)

**^1^** Department of Postharvest and Food Sciences, ARO, the Volcani Center, Bet Dagan 50250, Israel

^2^ Department of Plant Pathology and Weed Research, ARO, the Volcani Center, Bet Dagan 50250, Israel

^3^ Department of Plant Pathology and Weed Research, the Volcani Center, Newe-Yaar Research Center, Israel.

^4^ [Department of Plant Pathology and Microbiology](http://departments.agri.huji.ac.il/plantpath/), [the Robert H. Smith Faculty of Agriculture, Food and Environment ,](http://www.agri.huji.ac.il/) [the Hebrew University of Jerusalem](http://www.huji.ac.il/), Israel.

^5^ U.S. Department of Agriculture-Agricultural Research Service (USDA-ARS), Appalachian Fruit Research Station, WV, USA.

† Equal contributors.

**Table 4**. Summary of differential expressed genes in M. fructicola interaction with P. digitatum and interaction with fruit ( *p* < 0.05) involved in transport (transmembrane GO:055085), ion (GO:006811), carbohydrate (GO:008643), lipid (GO:006869), amino acid transport (GO:006865).

|  |  |  |  |  |  |
| --- | --- | --- | --- | --- | --- |
| Standard name | Systematic name | contig | Description | log FC Pdig | log FC fruit |
| **Transmembrane transport (GO:055085)** | | | | | |
| GAP1 | YKR039W | comp1140_c0 | General amino acid permease; Gap1p senses the presence of amino acid substrates to regulate localization to the plasma membrane when needed | 4.3 | 4.2 |
| ITR2 | YOL103W | comp5062_c0 | Myo-inositol transporter with strong similarity to the major myo-inositol transporter Itr1p, member of the sugar transporter superfamily; expressed constitutively | 2.8 | 3.8 |
| OPT2 | YPR194C | comp5533_c0 | Oligopeptide transporter; member of the OPT family, with potential orthologs in S. pombe and C. albicans; also plays a role in formation of mature vacuoles | 3.2 | 3.5 |
| HXT3 | YDR345C | comp6779_c0 | Low affinity glucose transporter of the major facilitator superfamily, expression is induced in low or high glucose conditions | 5 | 4.6 |
| GIT1 | YCR098C | comp8775_c0 | Plasma membrane permease, mediates uptake of glycerophosphoinositol and glycerophosphocholine as sources of the nutrients inositol and phosphate; expression and transport rate are regulated by phosphate and inositol availability | 3.9 | 4.0 |
| SNF3 | YDL194W | comp928_c0 | Plasma membrane low glucose sensor that regulates glucose transport; contains 12 predicted transmembrane segments and a long C-terminal tail required for induction of hexose transporters; also senses fructose and mannose; similar to Rgt2p | 4.3 |  |
| HXT4 | YHR092C | comp5468_c0 | High-affinity glucose transporter of the major facilitator superfamily, expression is induced by low levels of glucose and repressed by high levels of glucose | 2.7 |  |
| PHO84 | YML123C | comp1288_c0 | High-affinity inorganic phosphate (Pi) transporter and low-affinity manganese transporter; regulated by Pho4p and Spt7p; mutation confers resistance to arsenate; | 3.4 |  |
| TPO1 | YLL028W | comp2555_c0 | Polyamine transporter that recognizes spermine, putrescine, and spermidine; catalyzes uptake of polyamines at alkaline pH and excretion at acidic pH; phosphorylation enhances activity and sorting to the plasma membrane | 3.6 |  |
| HXT6 | YDR343C | comp2682_c0 | High-affinity glucose transporter of the major facilitator superfamily, nearly identical to Hxt7p, expressed at high basal levels relative to other HXTs, repression of expression by high glucose requires SNF3 | 2.5 |  |
| VBA5 | YKR105C | comp3135_c0 | Putative transporter of the Major Facilitator Superfamily (MFS); proposed role as a basic amino acid permease based on phylogeny | 3.0 |  |
| AGP3 | YFL055W | comp5793_c0 | Low-affinity amino acid permease, may act to supply the cell with amino acids as nitrogen source in nitrogen- poor conditions; transcription is induced under conditions of sulfur limitation; plays a role in regulating Ty1 transposition | 2.5 |  |
| CAN | YEL063C | comp1249_c0 | Plasma membrane arginine permease, requires phosphatidyl ethanolamine (PE) for localization, exclusively associated with lipid rafts; mutation confers canavanine resistance | 2.7 |  |
| PMC1 | YGL006W | comp1005_c0 | Vacuolar Ca2+ ATPase involved in depleting cytosol of Ca2+ ions; prevents growth inhibition by activation of calcineurin in the presence of elevated concentrations of calcium; similar to mammalian PMCA1a | 2.7 |  |
| STL1 | YDR536W | comp334_c0 | Glycerol proton symporter of the plasma membrane, subject to glucose-induced inactivation, strongly but transiently induced when cells are subjected to osmotic shock | -2.8 |  |
| HSP78 | YDR258C | comp39_c0 | Oligomeric mitochondrial matrix chaperone that cooperates with Ssc1p in mitochondrial thermotolerance after heat shock; able to prevent the aggregation of misfolded proteins as well as resolubilize protein aggregates | -2.2 | -3.1 |
| FRE1 | YLR214W | comp189_c0 | Ferric reductase and cupric reductase, reduces siderophore-bound iron and oxidized copper prior to uptake by transporters; expression induced by low copper and iron levels | -5.2 | -5.3 |
| ZRT2 | YLR130C | comp2025_c0 | Low-affinity zinc transporter of the plasma membrane; transcription is induced under low-zinc conditions by the Zap1p transcription factor | -2.8 | 5.9 |
| SIT1 | YEL065W | comp7737_c0 | Ferrioxamine B transporter, member of the ARN family of transporters that specifically recognize siderophore-iron chelates; transcription is induced during iron deprivation and diauxic shift; potentially phosphorylated by Cdc28p |  | 4.3 |
| HXT1 | YHR094C | comp3730_c0 | Low-affinity glucose transporter of the major facilitator superfamily, expression is induced by Hxk2p in the presence of glucose and repressed by Rgt1p when glucose is limiting |  | 3.5 |
|  |  |  |  |  |  |
| ZRT3 | YKL175W | comp2107_c0 | Vacuolar membrane zinc transporter, transports zinc from storage in the vacuole to the cytoplasm when needed; transcription is induced under conditions of zinc |  | 3.9 |
| SUL1 | YBR294W | comp3662_c0 | High affinity sulfate permease of the SulP anion transporter family; sulfate uptake is mediated by specific sulfate transporters Sul1p and Sul2p, which control the concentration of endogenous activated sulfate intermediates |  | 3.0 |
| ITR1 | YDR497C | comp443_c0 | Myo-inositol transporter with strong similarity to the minor myo-inositol transporter Itr2p, member of the sugar transporter superfamily; expression is repressed by inositol and choline via Opi1p and derepressed via Ino2p and Ino4p |  | -3.1 |
| STL1 | YDR536W | comp334_c0 | Glycerol proton symporter of the plasma membrane, subject to glucose-induced inactivation, strongly but transiently induced when cells are subjected to osmotic shock |  | -3.6 |
| SSC1 | YJR045C | comp93_c0 | Hsp70 family ATPase, constituent of the import motor component of the Translocase of the Inner Mitochondrial membrane (TIM23 complex); involved in protein translocation and folding; subunit of SceI endonuclease | - | -3.2 |
| HSP60 | YLR259C | comp64_c0 | Tetradecameric mitochondrial chaperonin required for ATP-dependent folding of precursor polypeptides and complex assembly; prevents aggregation and mediates protein refolding after heat shock; role in mtDNA transmission; phosphorylated | - | -3.5 |
|  | NA | comp9973_c0 |  |  | 4.5 |
|  | NA | comp5274_c0 |  |  | 4.6 |
|  | NA | comp3905_c0 |  |  | 3.6 |
|  |  |  | **ion transport (GO:006811)** |  |  |
| SUL1 | YBR294W | comp3662_c0 | High affinity sulfate permease of the SulP anion transporter family; sulfate uptake is mediated by specific sulfate transporters Sul1p and Sul2p, which control the concentration of endogenous activated sulfate intermediates | - | 3.5 |
| GIT1 | YCR098C | comp8775_c0 | Plasma membrane permease, mediates uptake of glycerophosphoinositol and glycerophosphocholine as sources of the nutrients inositol and phosphate; expression and transport rate are regulated by phosphate and inositol availability | 3.9 | 4.0 |
| mep2 | YNL142W | comp8962_c0 | Ammonium permease involved in regulation of pseudohyphal growth; belongs to a ubiquitous family of cytoplasmic membrane proteins that transport only ammonium (NH4+); expression is under the nitrogen catabolite repression regulation | 3.1 | - |
| PMC1 | YGL006W | comp1005_c0 | Vacuolar Ca2+ ATPase involved in depleting cytosol of Ca2+ ions; prevents growth inhibition by activation of calcineurin in the presence of elevated concentrations of calcium; similar to mammalian PMCA1a | 2.7 | - |
| PHO84 | YML123C | comp1288_c0 | High-affinity inorganic phosphate (Pi) transporter and low-affinity manganese transporter; regulated by Pho4p and Spt7p; mutation confers resistance to arsenate; exit from the ER during maturation requires Pho86p | 3.4 | - |
| FRE1 | YLR214W | comp189_c0 | Ferric reductase and cupric reductase, reduces siderophore-bound iron and oxidized copper prior to uptake by transporters; expression induced by low copper and iron levels | -5.3 | -5.3 |
| ZRT2 | YLR130C | comp2025_c0 | Low-affinity zinc transporter of the plasma membrane; transcription is induced under low-zinc conditions by the Zap1p transcription factor | -2.8 | 5.9 |
| FET3 | YMR058W | comp1589_c0 | Ferro-O2-oxidoreductase required for high-affinity iron uptake and involved in mediating resistance to copper ion toxicity, belongs to class of integral membrane multicopper oxidases | - | 2.7 |
| FTR1 | YER145C | comp4109_c0 | High affinity iron permease involved in the transport of iron across the plasma membrane; forms complex with Fet3p; expression is regulated by iron | - | 5.7 |
| SIT1 | YEL065W | comp7737_c0 | Ferrioxamine B transporter, member of the ARN family of transporters that specifically recognize siderophore-iron chelates; transcription is induced during iron deprivation and diauxic shift; potentially phosphorylated by Cdc28p | - | 4.3 |
| ZRT3 | YKL175W | comp2107_c0 | Vacuolar membrane zinc transporter, transports zinc from storage in the vacuole to the cytoplasm when needed; transcription is induced under conditions of zinc deficiency | - | 3.9 |
|  |  |  |  |  |  |
|  |  |  | **carbohydrate transport (GO:008643)** |  |  |
| SNF3 | YDL194W | comp928_c0 | Plasma membrane low glucose sensor that regulates glucose transport; contains 12 predicted transmembrane segments and a long C-terminal tail required for induction of hexose transporters; also senses fructose and mannose; similar to Rgt2p | 4.3 | - |
| HXT6 | YDR343C | comp2682_c0 | High-affinity glucose transporter of the major facilitator superfamily, nearly identical to Hxt7p, expressed at high basal levels relative to other HXTs, repression of expression by high glucose requires SNF3 | 2.7 | - |
| HXT4 | YHR092C | comp5468_c0 | High-affinity glucose transporter of the major facilitator superfamily, expression is induced by low levels of glucose and repressed by high levels of glucose | 2.7 | - |
| HXT1 | YHR094C | comp3730_c0 | Low-affinity glucose transporter of the major facilitator superfamily, expression is induced by Hxk2p in the presence of glucose and repressed by Rgt1p when glucose is limiting | - | 3.5 |
| STL1 | YDR536W | comp334_c0 | Glycerol proton symporter of the plasma membrane, subject to glucose-induced inactivation, strongly but transiently induced when cells are subjected to osmotic shock | -3.6 | -2.5 |
|  |  |  | **lipid transport (GO:006869**) |  |  |
| RSB1 | YOR049C | comp1364_c0 | Suppressor of sphingoid long chain base (LCB) sensitivity of an LCB-lyase mutation; putative integral membrane transporter or flippase that may transport LCBs from the cytoplasmic side toward the extracytoplasmic side of the membrane | 3.1 | - |
|  |  |  | **amino acid transport (GO:006865)** |  |  |
| VBA3 | YCL069W | comp4427_c0 | Permease of basic amino acids in the vacuolar membrane | 2.5 | - |
| AGP3 | YFL055W | comp5793_c0 | Low-affinity amino acid permease, may act to supply the cell with amino acids as nitrogen source in nitrogen- poor conditions; transcription is induced under conditions of sulfur limitation; plays a role in regulating Ty1 transposition | 2.5 | - |
| GAP1 | YKR039W | comp1140_c0 | General amino acid permease; Gap1p senses the presence of amino acid substrates to regulate localization to the plasma membrane when needed | 4.3 | 4.2 |
