# Supplementary material for: De-novo assembly and characterization of the transcriptome of Metschnikowia fructicola reveals differences in gene expression following interaction with Penicillium digitatum and grapefruit peel
Source: BMC Genomics. 2013 Mar 12;14:168. doi: 10.1186/1471-2164-14-168 (PMC3608080; doi:10.1186/1471-2164-14-168)
Supplement: Additional file 7 — Summary of differential expressed genes in Metschnikowia fructicola interaction with Penicillium digitatum and interaction with fruit (p < 0.05) involved in metabolic (lipid GO:006629), (vitamin GO:006766), (cofactor GO:051186), (cellular amino acid GO:006520) processes. [file 1471-2164-14-168-S7.docx]

***De-novo* assembly and characterization of the transcriptome of *Metschnikowia fructicola* reveals differences in gene expression following interaction with *Penicillium digitatum* and grapefruit peel**

**Vera Hershkovitz^1,^** **^†^**

Email: vhershko@agri.gov.il

**Noa Sela^2, †^**

Email: [noa@agri.gov.il](mailto:noa@agri.gov.il)

**Leena Taha-Salaime^1,3,4^**

Email: [leena.taha@mail.huji.ac.il](mailto:leena.taha@mail.huji.ac.il)

**Jia Liu^5^**

Email:Jia.Liu@ARS.USDA.GOV

**Ginat Rafael^1^**

Email: [pongie@volcani.agri.gov.il](mailto:pongie@volcani.agri.gov.il)

**Clarita Kessler^1^**

Email: [clarita.bendayan@gmail.com](mailto:clarita.bendayan@gmail.com)

**Radi Aly^3^**

Email: [radi@volcani.agri.gov.il](mailto:radi@volcani.agri.gov.il)

**Maggie Levy^4^**

Email: [levym@agri.huji.ac.il](mailto:levym@agri.huji.ac.il)

**Michael Wisniewski^5^**

Email: Michael.Wisniewski@ARS.USDA.GOV

**Samir Droby^1*^**

* Corresponding author

Email: samird[@volcani.agri.gov.il](mailto:samird@volcani.agri.gov.il)

**^1^** Department of Postharvest and Food Sciences, ARO, the Volcani Center, Bet Dagan 50250, Israel

^2^ Department of Plant Pathology and Weed Research, ARO, the Volcani Center, Bet Dagan 50250, Israel

^3^ Department of Plant Pathology and Weed Research, the Volcani Center, Newe-Yaar Research Center, Israel.

^4^ [Department of Plant Pathology and Microbiology](http://departments.agri.huji.ac.il/plantpath/), [the Robert H. Smith Faculty of Agriculture, Food and Environment ,](http://www.agri.huji.ac.il/) [the Hebrew University of Jerusalem](http://www.huji.ac.il/), Israel.

^5^ U.S. Department of Agriculture-Agricultural Research Service (USDA-ARS), Appalachian Fruit Research Station, WV, USA.

† Equal contributors.

**Table S5**. Summary of differential expressed genes in M. fructicola interaction with P. digitatum and interaction with fruit ( p < 0.05) involved in metabolic (lipid GO:006629), (vitamin GO:006766), (cofactor GO:051186), (cellular amino acid GO:006520) processes.

|  |  |  |  |  |  |
| --- | --- | --- | --- | --- | --- |
| Standard name | Systematic name | contig | Description | log FC Pdig | log FC fruit |
| **Lipid metabolic processes (GO:006629)** | | | | | |
| ISC1 | YER019W | comp2610_c0 | Mitochondrial membrane localized inositol phosphosphingolipid phospholipase C, hydrolyzes complex sphingolipids to produce ceramide; activated by phosphatidylserine, cardiolipin, and phosphatidylglycerol; mediates Na+ and Li+ halotolerance | -2.5 |  |
| AYR1 | YIL124W | comp3427_c0 | NADPH-dependent 1-acyl dihydroxyacetone phosphate reductase found in lipid particles, ER, and mitochondrial outer membrane; involved in phosphatidic acid biosynthesis; required for spore germination; capable of metabolizing steroid hormones | 3.9 |  |
| GPI16 | YHR188C | comp5455_c0 | Transmembrane protein subunit of the glycosylphosphatidylinositol transamidase complex that adds GPIs to newly synthesized proteins; human PIG-Tp homolog | -4.8 |  |
| ERG1 | YGR175C | comp2062_c0 | Squalene epoxidase, catalyzes the epoxidation of squalene to 2,3-oxidosqualene; plays an essential role in the ergosterol-biosynthesis pathway and is the specific target of the antifungal drug terbinafine | - | 3.1 |
| ERG11 | YHR007C | comp680_c0 | Lanosterol 14-alpha-demethylase, catalyzes the C-14 demethylation of lanosterol to form 4,4''-dimethyl cholesta-8,14,24-triene-3-beta-ol in the ergosterol biosynthesis pathway; member of the cytochrome P450 family | - | 3.4 |
| ERG5 | YMR015C | comp9413_c0 | C-22 sterol desaturase, a cytochrome P450 enzyme that catalyzes the formation of the C-22(23) double bond in the sterol side chain in ergosterol biosynthesis; may be a target of azole antifungal drugs | - | 2.6 |
| DAP1 | YPL170W | comp1700_c0 | Heme-binding protein involved in regulation of cytochrome P450 protein Erg11p; damage response protein, related to mammalian membrane progesterone receptors; mutations lead to defects in telomeres, mitochondria, and sterol synthesis | - | 3.0 |
|  |  |  | **Vitamin metabolic processes (GO:006766)** |  |  |
| THI4 | YGR144W | comp5488_c0 | Thiazole synthase, catalyzes formation of a thiazole intermediate during thiamine biosynthesis; required for mitochondrial genome stability in response to DNA damaging agents |  | 3.2 |
| FMS1 | YMR020W | comp9998_c0 | Polyamine oxidase, converts spermine to spermidine, which is required for the essential hypusination modification of translation factor eIF-5A; also involved in pantothenic acid biosynthesis | 3.5 | - |
| THI11 | YJR156C | comp1015_c0 | Protein involved in synthesis of the thiamine precursor hydroxymethylpyrimidine (HMP); member of a subtelomeric gene family including THI5, THI11, THI12, and THI13 | -2.8 | - |
|  |  |  | **Cofactor metabolic process (GO:051186)** |  |  |
| HEM13 | YDR044W | comp1363_c0 | Coproporphyrinogen III oxidase, an oxygen requiring enzyme that catalyzes the sixth step in the heme biosynthetic pathway; transcription is repressed by oxygen and heme (via Rox1p and Hap1p) | - | 3.9 |
| BNA2 | YJR078W | comp2126_c0 | Putative tryptophan 2,3-dioxygenase or indoleamine 2,3-dioxygenase, required for de novo biosynthesis of NAD from tryptophan via kynurenine; interacts genetically with telomere capping gene CDC13; regulated by Hst1p and Aftp | - | 3.3 |
| ALD4 | YOR374W | comp947_c0 | Mitochondrial aldehyde dehydrogenase, required for growth on ethanol and conversion of acetaldehyde to acetate; phosphorylated; activity is K+ dependent; utilizes NADP+ or NAD+ equally as coenzymes; expression is glucose repressed | 2.7 | - |
| FMS1 | YMR020W | comp9998_c0 | Polyamine oxidase, converts spermine to spermidine, which is required for the essential hypusination modification of translation factor eIF-5A; also involved in pantothenic acid biosynthesis | 3.5 | - |
| ADH2 | YMR303C | comp31_c0 | Glucose-repressible alcohol dehydrogenase II, catalyzes the conversion of ethanol to acetaldehyde; involved in the production of certain carboxylate esters | -3.2 | - |
| MDH1 | YKL085W | comp84_c0 | Mitochondrial malate dehydrogenase, catalyzes interconversion of malate and oxaloacetate; involved in the tricarboxylic acid (TCA) cycle; phosphorylated | -4.1 | -4.1 |
|  |  |  | **Cellular amino acid metabolic processes (GO:006520)** |  |  |
| AGP3 | YFL055W | comp5793_c0 | Low-affinity amino acid permease, may act to supply the cell with amino acids as nitrogen source in nitrogen- poor conditions; transcription is induced under conditions of sulfur limitation; plays a role in regulating Ty1 transposition | 2.5 | - |
| GAP1 | YKR039W | comp1140_c0 | General amino acid permease; Gap1p senses the presence of amino acid substrates to regulate localization to the plasma membrane when needed | 4.3 | 4.2 |
| CPA2 | YJR109C | comp4738_c0 | Large subunit of carbamoyl phosphate synthetase, which catalyzes a step in the synthesis of citrulline, an arginine precursor | 3.2 | - |
| MAE1 | YKL029C | comp10104_c0 | Mitochondrial malic enzyme, catalyzes the oxidative decarboxylation of malate to pyruvate, which is a key intermediate in sugar metabolism and a precursor for synthesis of several amino acids | 3.3 | - |
| CAR2 | YLR438W | comp5796_c0 | L-ornithine transaminase (OTAse), catalyzes the second step of arginine degradation, expression is dually- regulated by allophanate induction and a specific arginine induction process; not nitrogen catabolite repression sensitive | 2.7 | - |
| FMS1 | YMR020W | comp9998_c0 | Polyamine oxidase, converts spermine to spermidine, which is required for the essential hypusination modification of translation factor eIF-5A; also involved in pantothenic acid biosynthesis | 3.5 | - |
| ADH2 | YMR303C | comp31_c0 | Glucose-repressible alcohol dehydrogenase II, catalyzes the conversion of ethanol to acetaldehyde; involved in the production of certain carboxylate esters; regulated by ADR1 | -3.2 | - |
| CHA1 | YCL064C | comp6609_c0 | Catabolic L-serine (L-threonine) deaminase, catalyzes the degradation of both L-serine and L- threonine; required to use serine or threonine as the sole nitrogen source, transcriptionally induced by serine and threonine | - | 3.5 |
| LAP3 | YNL239W | comp5339_c0 | Cysteine aminopeptidase with homocysteine- thiolactonase activity; protects cells against homocysteine toxicity; has bleomycin hydrolase activity in vitro | - | 4.7 |
| ARO1 | YDR127W | comp8321_c0 | Pentafunctional aroma protein, catalyzes steps 2 through 6 in the biosynthesis of chorismate, which is a precursor to aromatic amino acids [ | - | 5.8 |
| LIA1 | YJR070C | comp4922_c0 | Deoxyhypusine hydroxylase, a HEAT-repeat containing metalloenzyme that catalyzes hypusine formation; binds to and is required for the modification of Hyp2p (eIF5A); | - | 3.2 |
| PDC1 | YLR044C | comp236_c0 | Major of three pyruvate decarboxylase isozymes, key enzyme in alcoholic fermentation, decarboxylates pyruvate to acetaldehyde; subject to glucose-, ethanol-, and autoregulation; involved in amino acid catabolism | - | 4.2 |
| ALD3 | YMR169C | comp4008_c0 | Cytoplasmic aldehyde dehydrogenase, involved in beta-alanine synthesis; uses NAD+ as the preferred coenzyme; very similar to Ald2p; expression is induced by stress and repressed by glucose | - | -3.8 |
| OXP1 | YKL215C | comp4643_c0 | 5-oxoprolinase; enzyme is ATP-dependent and functions as a dimer; similar to mouse Oplah gene; green fluorescent protein (GFP)-fusion protein localizes to the cytoplasm | - | -4.4 |
| CAR1 | YPL111W | comp776_c0 | Arginase, responsible for arginine degradation, expression responds to both induction by arginine and nitrogen catabolite repression; disruption enhances freeze tolerance | - | -4.1 |
| GCV2 | YMR189W | comp477_c0 | P subunit of the mitochondrial glycine decarboxylase complex, required for the catabolism of glycine to 5,10-methylene-THF; expression is regulated by levels of 5,10-methylene-THF in the cytoplasm | - | -4.4 |
