# Supplementary material for: De-novo assembly and characterization of the transcriptome of Metschnikowia fructicola reveals differences in gene expression following interaction with Penicillium digitatum and grapefruit peel
Source: BMC Genomics. 2013 Mar 12;14:168. doi: 10.1186/1471-2164-14-168 (PMC3608080; doi:10.1186/1471-2164-14-168)
Supplement: Additional file 8 — The primer sequences used in qPCR. [file 1471-2164-14-168-S8.docx]

***De-novo* assembly and characterization of the transcriptome of *Metschnikowia fructicola* reveals differences in gene expression following interaction with *Penicillium digitatum* and grapefruit peel**

**Vera Hershkovitz^1,^** **^†^**

Email: vhershko@agri.gov.il

**Noa Sela^2, †^**

Email: [noa@agri.gov.il](mailto:noa@agri.gov.il)

**Leena Taha-Salaime^1,3,4^**

Email: [leena.taha@mail.huji.ac.il](mailto:leena.taha@mail.huji.ac.il)

**Jia Liu^5^**

Email:Jia.Liu@ARS.USDA.GOV

**Ginat Rafael^1^**

Email: [pongie@volcani.agri.gov.il](mailto:pongie@volcani.agri.gov.il)

**Clarita Kessler^1^**

Email: [clarita.bendayan@gmail.com](mailto:clarita.bendayan@gmail.com)

**Radi Aly^3^**

Email: [radi@volcani.agri.gov.il](mailto:radi@volcani.agri.gov.il)

**Maggie Levy^4^**

Email: [levym@agri.huji.ac.il](mailto:levym@agri.huji.ac.il)

**Michael Wisniewski^5^**

Email: Michael.Wisniewski@ARS.USDA.GOV

**Samir Droby^1*^**

* Corresponding author

Email: samird[@volcani.agri.gov.il](mailto:samird@volcani.agri.gov.il)

**^1^** Department of Postharvest and Food Sciences, ARO, the Volcani Center, Bet Dagan 50250, Israel

^2^ Department of Plant Pathology and Weed Research, ARO, the Volcani Center, Bet Dagan 50250, Israel

^3^ Department of Plant Pathology and Weed Research, the Volcani Center, Newe-Yaar Research Center, Israel.

^4^ [Department of Plant Pathology and Microbiology](http://departments.agri.huji.ac.il/plantpath/), [the Robert H. Smith Faculty of Agriculture, Food and Environment ,](http://www.agri.huji.ac.il/) [the Hebrew University of Jerusalem](http://www.huji.ac.il/), Israel.

^5^ U.S. Department of Agriculture-Agricultural Research Service (USDA-ARS), Appalachian Fruit Research Station, WV, USA.

† Equal contributors.

**Table 1.**  Specific primers used for transcription analysis by qPCR mRNA

| Primer reverse (5’-3’) | Primer forward (5’-3’) | | Gene |
| --- | --- | --- | --- |
| ACCATTTGTCGCCCAATTCCGAAC | AGATCAGCCCGCGTCATTCCTATT | HSP40 (cont13) | |
| ATTCCTATGAAGGCCGGAAATGGC | ACTTGCTTCACGGCCATCAATTCG | MEKK  (cont 4269) | |
| CTTCTTGCGTAAACGGCGTCTCAA | ATCTTCAACGCACAAACCTCACGC | Ser/Thr kinase  (cont2116) | |
| AACTGGATTTCCGGTTTGAACGGC | ACAGTACAAGCCACCAACGGAGAT | GLU  (cont1118) | |
| TACAGTAACCCGGGCCAGTTCAAA | TATAGCACCTTGCGCAGTTCAGGT | ABC transporter (cont6782) | |
| TACAGTAACCCGGGCCAGTTCAAA | TATAGCACCTTGCGCAGTTCAGGT | TF STB5  (cont7125) | |
| TGTCACTTAATCCAGGCGACGAGT | GGGATTACATCAACAACGCTGCCA | SOD1  (cont513) | |
| TGCATCACTCTCGAAGCCGTACTT | TCTCTAACATGTGCTCCAAGGCGT | CHI HQ113461 | |
| GAGTTGTAAGTGGTTTGGTCG | CCTGAGGAACACCCAGTCTT | Actin, AJ745127 | |
